# Extranodal Extension in Bilateral Cervical Metastases: A predictor of Undesirable Survival Outcomes despite Aggressive Salvage Treatment in Oral Cancer Patients

**DOI:** 10.7150/jca.60152

**Published:** 2021-08-03

**Authors:** Weijin Gao, Yuhua Hu, Dan Zhu, Xiaoguang Li, Bing Guo, Yi Shen, Chunyue Ma, Juan Du

**Affiliations:** 1 Department of Dermatology, Huashan Hospital affiliated by Fudan University, No.12, Wulumuqi Middle Road, Shanghai, China.; 2 Department of Maxillofacial - Head & Neck Oncology, 9th People's Hospital, Shanghai Jiao Tong University School of Medicine, National Research Center for Oral Diseases, Shanghai Key Laboratory of Stomatology, No. 639, Zhi Zao Ju Road, Shanghai 200011, China.; 3 Department of Oral Pathology, 9th People's Hospital, Shanghai Jiao Tong University School of Medicine, No. 639, Zhi Zao Ju Road, Shanghai 200011, China.; 4 Department of Radiology, 9th People's Hospital, Shanghai Jiao Tong University School of Medicine, No. 639, Zhi Zao Ju Road, Shanghai 200011, China.; 5 Department of Oral and Maxillofacial Surgery, The First Affiliated Hospital of Wenzhou Medical University, Wenzhou 325000, Zhejiang, China.

**Keywords:** bilateral, extranodal extension, cervical metastasis, oral cancer, surgery, survival

## Abstract

**Objectives:** Despite the inclusion of extranodal extension (ENE) in the recent staging system, the presence of ENE alone is not sufficient to depict all clinical situations, as ENE is frequently found in multiple nodes. Thus, the purpose of this study was to evaluate the surgery-based treatment outcomes and clinicopathological features of oral cavity squamous cell carcinoma (OCSCC) patients with ENE found in bilateral multiple cervical metastases.

**Materials and methods:** A retrospective single-institutional study of OCSCC patients with bilateral ENE nodes was performed from January 2011 to December 2018. OCSCC patients of different admission statuses (with primary lesions (PL), recurrent lesions (RL) and isolated neck metastases (INM)) were included for subgroup comparisons. All patients received surgical treatment with/without adjuvant therapies and had complete follow-up data. Disease-free survival (DFS) was regarded as the main outcome. Time-to-relapse data were also collected for comparison.

**Results:** A total of 128 patients were included, of whom 97 (75.8%) were male. The mean follow-up period reached 15 months. Among the patients, 85 (66.4%) were treated for PLs, followed by 26 (20.3%) treated for RLs after failed prior therapy and 17 (13.3%) treated for INMs (concurrent or sequential). The DFS rate was merely 35.2%. Treatment-related factors such as surgical margin (p=0.003), postoperative adjuvant therapy (p=0.014) and perioperative complications (p=0.036) were significantly associated with patient outcomes. In addition, oral lesion-related variables such as oral subsites (p=0.037), T classification (p=0.026) and skull base involvement (p=0.040) were indicators of a worse prognosis. For bilateral ENE features, ENE subclassification (p=0.036), maximum size of ENE nodes (p=0.039) and arterial nodal encasement (p=0.025) tended to predict the surgery-based treatment outcomes of these patients.

**Conclusions:** Bilateral cervical metastases with ENE features, though uncommon, are a serious regional burden, and these patients have lower-than-expected treatment outcomes, especially those with RLs or INMs. A fairly large number of OCSCC patients with advanced oral lesions gain little benefit from intensified salvage surgical treatment. Such treatment should instead be offered to select patients with smaller bilateral ENE nodes (<3 cm) and those with lower ENE subclassifications and no arterial nodal encasement.

## Introduction

The presence of metastatic lymph nodes (MLNs) has widely been accepted as an important prognostic factor for patients diagnosed with oral cavity squamous cell carcinoma (OCSCC) 1,2. Additionally, it has been found in many studies that approximately 30-50% of primary OCSCC patients have nodal involvement, leading to a significant reduction in locoregional control and overall survival (OS) 3-5. Such a high incidence of MLN has initiated an intense wave of investigations into its clinical implications in OCSCC. Thus, a large number of MLN indices, such as total nodal volume 6-7, nodal necrosis 8 and lymph node ratio (LNR) 9-10, have been proposed to reflect the seriousness of metastatic burdens. Apart from these indices, it was not until 2013 that the presence of extranodal extension (ENE) or spread was first proposed as one of the dichotomized criteria, along with the size of the MLN, for determining the cervical stages in head and neck cancers 11-13. Currently, the importance of this factor has found general acceptance with increasing clinical evidence regarding its relation to prognosis. The incorporation of ENE in the new Tumor, Node, Metastasis (TNM) Staging System also highlights its role in the stratification of high-risk patients for more aggressive treatment regimens 11, which is routinely applied when evaluating clinical care for OCSCC patients.

Theoretically speaking, ENE is commonly defined as metastatic cancer cells that extend through the lymph node capsule into the surrounding connective tissues 14. Such characteristics were previously found to be closely relevant to regional nodal recurrence and distant metastasis 15-16. Compared with conventional MLNs, MLNs with ENEs are more likely to be found in those with advanced primary lesions because their synergistic effect contributes to poorer treatment outcomes 17-19. Shaw also confirmed this claim, with reported OS rates of 65%, 52% and 23% for node-negative, node-positive (ENE-negative) and node-positive (ENE-positive) patients, respectively 17. Despite these findings, the full influence of ENE has not yet been elucidated, as most studies still consider ENE as a single pathologic event when grading patients with OCSCC 20-21. Nevertheless, few reports have focused on the clinical dilemmas for those with bilateral multilevel MLNs with ENE features. The development of such bilateral ENE nodes varies according to the different locations of diseases, prior treatment and adjuvant therapies. Accordingly, the treatment rationale and prognoses for patients with bilateral ENE nodes may differ depending on specific locoregional conditions. In addition, the efficacy of surgery-based therapies, which are usually the first option against OCSCC for these patients, has been questioned due to likely increased odds of relapse or distant metastasis. Thus, the aim of this retrospective study was to tentatively resolve these dual concerns in terms of the specific ENE nodal features and the prognosis of OCSCC patients with bilateral ENE burdens. Unilateral ENE burdens, which included ipsilateral or contralateral ENE nodes in OCSCC patients were also compared for analyses. Those who might benefit from aggressive treatment are also discussed for possible subclassification.

## Methods and materials

### Study population and inclusion criteria

This was a retrospective single-institutional study that included OCSCC patients with either ipsilateral/contralateral ENE nodes, or with bilateral ENE ones, who received surgery with/without adjuvant therapies between January 2011 and December 2018. The follow-up duration was calculated from dates of surgical treatment in our institution until death/last follow-up visits in months. Due to the retrospective nature of this study, approval was granted by the independent institutional ethics committee of our hospital (approval number: SH9H-2021-TK165). In addition, written consent was obtained from the patients whose clinical images are shown in this study.

With regard to the study aim, the inclusion criteria for this study were as follows: 1) patients with primary or recurrent OCSCCs or with isolated neck metastases (concurrently or sequentially) after failed watchful observations for early-stage OCSCC patients; 2) patients with pathological ENE evidence found in either ipsilateral/contralateral or bilateral cervical nodes; 3) patients surgically treated with curative rather than palliative intent; and 4) patients without distant metastases. The candidates included in this study met all these criteria. The exclusion criteria were as follows: 1) patients with incomplete medical records or follow-up information; 2) patients treated with only adjuvant, non-surgical therapies (radiotherapy, radiochemotherapy or targeted/immunotherapies) and 3) patients without ENE nodes.

### Demographic information and prior treatment history

Demographic information (age, sex and smoking history) was directly collected from the chart database. History of prior and present treatment was also obtained to classify these OCSCC patients with either unilateral or bilateral ENE nodes into three distinct subgroups: primary lesion (PL), recurrent lesion (RL), isolated neck metastases (INM) groups. The RL group included patients with relapsed/secondary primary lesions, while the INM group included those with ipsilateral/contralateral or bilateral cervical metastases and the absence of oral primary lesion relapses after previous surgical treatment. Due to the complicated treatment regimens and disease statuses of these patients, the following statistics regarding these three groups were both collectively (between groups) and separately (i.e., within group) compared.

### Oral-cavity tumor characteristics

Information regarding the oral subsite, size and pathologic grades of OCSCC was collected, and the tumor (T) classification was recorded according to the 8^th^ edition of the American Joint Committee on Cancer (AJCC) system 22. In an attempt to further delineate the disease conditions of these OCSCC patients, along with nodal information, other special radiologic-pathologic characteristics of oral lesions, such as inseparable oral and cervical lesions, midline involvement, bone invasion, depth of invasion (DOI) and perineural invasion (PNI), were re-reviewed and included in this study. Oral lesions of the INM group were determined by previous treatment records and pathologic reviews. Human papillomavirus (HPV) detection, either P16 or HPV DNA tests, was performed in some cases with exophytic growth or with proximity to oropharyngeal anatomies. However, considering the generally low incidence of infection in OCSCC samples, HPV examinations were not routinely performed in our cohort. In addition, the preoperative radiologic suspicion of either ipsilateral/contralateral or bilateral ENE was verified by comparing the final pathologic results.

### Cervical metastases and ENE data

Since ENE was first incorporated into the AJCC classification in 2017, the pathological sections obtained before April 2018 (with descriptions of extranodal spread, extension, or surrounding-tissue invasion) were reviewed by two experienced pathologists (Y.H. and J.D). In addition, specific nodal information regarding the total number of metastatic lymph nodes, metastatic LNR, greatest dimensions of metastatic nodes, level of ipsilateral/contralateral or bilateral ENE, metastatic lymph node fusion (inseparable metastatic nodes), nodal necrosis, cutaneous, muscle or vascular invasion (including oncologic venous embolism), and even mandibular or skull base bone involvement was recorded for analysis. According to the new International Collaboration on Cancer Reporting (ICCR) recommendations, the grade of ENE was also determined for the different depths of extracapsular extension of MLNs, as minor ENE (ENEmi) for extension of up to 2 mm (≤2 mm) from the lymph node capsule and major ENE (ENEma) as extension of more than 2 mm (>2 mm), which always includes gross carcinogenic deposits in cervical soft tissues with blurred/without normal nodal architecture 23.

### Surgery and adjuvant treatment

Treatment regarding the extent of resection was indirectly reflected in the reconstructive parameters, such as flap sizes and types. For the treatment of ENE nodes, the aggressiveness of neck dissections was classified according to the level of involvement, such as supra-omohyoid neck dissection (SOND) from level I to III, extended SOND from level I to IV, and radical neck dissection (RND) from level I to V. The application of en bloc procedures (oral lesions resected together with cervical lymph nodal samples) was also analyzed. In addition, postoperative margin status was reported to describe the completeness of surgical resection, and postoperative complications were recorded based on the chart review.

Data regarding pre- and postoperative adjuvant therapies were also collected. For the RL group, the application of reirradiation was also analyzed for efficacy. Although no immune therapy was applied in the current cohort due to government approval and market access at that time, anti-epithelial growth factor receptor (anti-EGFR) therapies were used in selected cases. The criteria for using anti-EGFR therapies, though self-pay (uncovered by the medical insurances), were mostly based on positive sample results of EGFR. Admittedly, the economic status of different patients also influenced the choices of such targeted treatment in our study.

### Follow-up information

For follow-up, all patients returned to the outpatient clinic every 1-3 months during the first three years, 6-9 months during the fourth to fifth years, and annually thereafter. The total follow-up time was calculated until the last follow-up or event of death, irrespective of the cause. Disease-free survival (DFS) was counted as the main outcome for this study. In order to better describe the treatment efficacies, time-to-relapse (TTR) data were also collected. Representative cases were also presented.

### Statistical analysis

The statistical analyses were performed with SPSS version 23.0 software (IBM Corp., Armonk, NY). The primary endpoint of this study was DFS. Logistic regression was utilized to determine the relevant factors of ENE. Cumulative survival curves were plotted with the Kaplan-Meier method (log-rank test). The TTR data for univariate and multivariate analyses were also given. Additionally, univariate and multivariate proportional hazard Cox models were used to evaluate the prognostic factors.

## Results

### Patient population and treatment summary

In total, 501 patients (331 male and 170 female) with either ipsilateral/contralateral ENE nodes, and 128 patients (97 male and 31 female) with bilateral ENE nodes were included in this study. The demographics and prior medical history are listed in***Table 1 (bilateral) and Supplementary Table 1 (ipsilateral)***. According to different treatment groups, within the patients with ipsilateral/contralateral ENE nodes, most patients were with primary lesions (n=326, 65.1%), while for the bilateral ENE group, 85 (66.4%) patients were treated for primary lesions, followed by 26 (20.3%) for recurrent lesions after failed prior treatment. Within the entire study population, comorbidities such as cardiovascular diseases, diabetes, and strokes were identified in 304 (60.7%) and 54 (42.2%) patients, respectively. In the bilateral ENE group, approximately one-third (33.6%) of the patients received prior excisional operations, while in the ipsilateral/contralateral ENE group, 169 patients received prior surgeries as primary treatment. In the bilateral ENE group, en-bloc resection (continuous oral lesion resection and neck dissection) was performed in 73 (57.0%) patients, while noncontinuous (separated) resection was performed in 55 (43.0%) patients, which also included 17 (13.3%) patients in the INM group who had isolated bilateral metastatic nodes with ENE features. There were 20 (4.0%) and 12 (9.4%) patients with reports of positive surgical margins in either unilateral and bilateral ENE groups, of whom all received postoperative adjuvant treatment. For the bilateral ENE group, the ablative operations involved several anatomic subsites, which later required large pedicled or free flap coverage, with 92 (71.9%) patients receiving flap reconstructions with a flap length (skin) over 10 cm. In contrast, for the unilateral ENE group, primary closure or minor flaps (<10 cm) were applied in 375 (74.9%) patients, indicative of a relatively small wound burden for the latter. Within the bilateral ENE group, 67 (52.3%) developed minor or major perioperative complications, of whom the symptoms were exacerbated and resulted in death in 2 cases (carotid blowout and hepatic failure). Pulmonary infections (n=32, 25.0%) were also frequently found in these bilateral metastatic patients due to the high rate of prophylactic tracheotomy.

For the bilateral ENE group, postoperative radiotherapy was administered to 31 (24.2%) patients, while radiochemotherapy was administered to 52 (40.6%) patients. Targeted therapies (anti-EGFR) were mostly administered in combination with other adjuvant treatments to 30 (23.4%) patients. For the unilateral ENE group, postoperative radiotherapy and radiochemotherapy were administered to 310 (61.9%) and 100 (20.0%) patients, respectively. Anti-EGFR therapy were applied in 58 (11.6%) patients.

### Oral-cavity tumor characteristics

Within the bilateral ENE group, tongue (60, 46.9%) and floor of the mouth 34 (26.6%) were found to be the most frequently affected subsites in this series (***Table 2***). For the unilateral ENE group, a similar trend (tongue=201, 40.1%) was also noticed (***Supplementary Table 2***). According to the AJCC classification, most patients (63, 49.2%) in the bilateral ENE group were graded as having T3 disease (including those in the INM and RL groups). The average size of the oral lesions reached 4.88 cm, with most oral lesions (n=92, 71.8%) exceeding 4 cm. In addition, the DOIs of oral lesions (bilateral ENE group) were generally (n=115, 89.8%) larger than 10 mm, indicative of the disease seriousness. In consideration of the clinical status of bilateral MLNs, we found OCSCC lesions invading through the midline in 96 (75.0%) patients. A low HPV infection rate (2.3% and 2.8%) was found in either unilateral or bilateral ENE group, though the infection rate in almost 80% of the cases remained unknown. The coexistence of ENE and PNI was also found in 69 (53.9%) patients (bilateral ENE group). In addition, mandibular (or maxillary) osseous destructions were not rare (n=48, 37.5%), with 8 (6.3%) cases having oral lesions that reached the skull base in the bilateral ENE group, while such osseous destructions were only found in 93 (18.6%) patients in the unilateral ENE one.

### Ipsilateral/contralateral and bilateral ENE features

For the unilateral ENE group, most ENE nodes (n=393, 78.4%), according to the ICCR classifications, were classified as ENEmi, signaling a less aggressive nature (***Supplementary Table 3***). Most unilateral ENE patients were with ipsilateral (n=416, 83%) ENE nodes. In addition, the average sizes for ipsilateral/contralateral ENE nodes reached only 3.6±1.2 cm, with most found in the upper I-III levels (n=422, 84.2%). Arterial encasement of ENE nodes was merely found in 15 (3.0%) cases, while evidence of internal jugular vein embolism was found in 48 (9.6%) cases. The number ratio between ENE and all excised nodes were 0.19±0.27.

**Figure 1:**
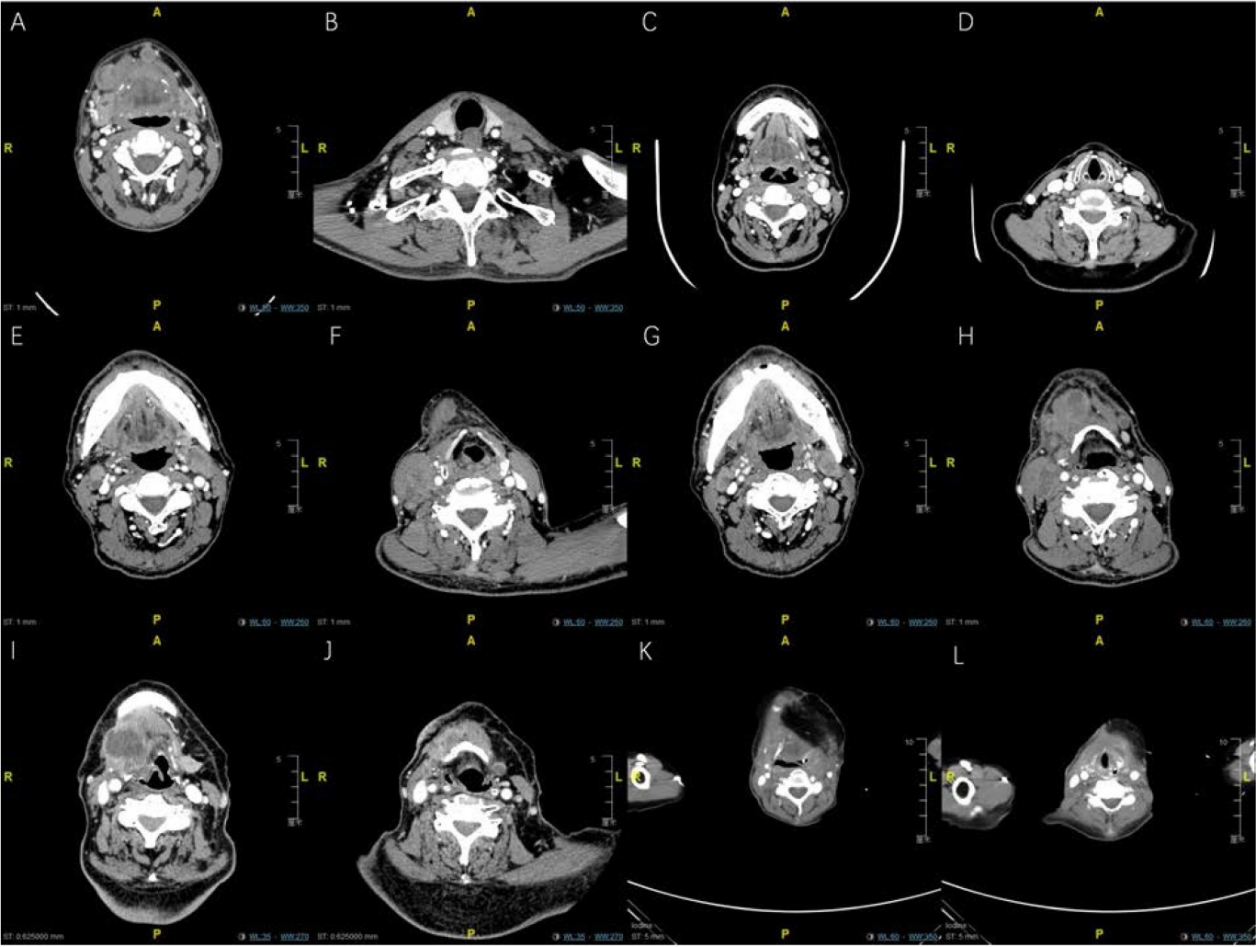
** Representative enhanced CT imaging for oral cancer patients with bilateral maENE nodes of various features (High resolution image is in the Supplementary material). A.** PL group: Case 1 with right-sided level Ib and left sided level Ia maENE metastases (obvious skin involvement). **B.** PL group: Case 1 with left-sided level IV ENE metastases (SCM invasion). **C.** PL group: Case 2 with miENE (extracapsular extention≤2mm) nodes in bilateral level Ib, and left-sided level IIa metastases (demonstrated by postoperative pathologic report). **D.** PL group: Case 2 with miENE nodes in left-sided level III showing multilevel ENE features. **E.** PL group: Case 3 with maENE (extracapsular extension>2 mm) nodes in bilateral level IIa, specifically around the carotid sheaths, invading both SCMs. **F.** PL group: Case 3 with right-sided maENE nodes causing ipsilateral carotid encasement and embolism of internal jugular vein. **G.** INM group: Case 4 with maENE nodes in bilateral level Ib. **H.** INM group: Case 4 with multiple maENE nodes in right-sided level IIa and IIb. Several maENE nodes were fused and inseparable, with necrotic changes. **I.** RL group: Case 5 with huge right-sided maENE necrotic node (level III) invading the hyoid bone. **J.** RL group: Case 5 with small residual left-sided miENE in the parahyoid region (demonstrated by postoperative pathologic report). **K.** RL group: Case 6 with recurrent parahyoid fused maENE nodes (right side) with close proximity with the prior reconstructed flap. **L.** RL group: Case 6 with recurrent parahyoid maENE nodes (left side) underneath the prior reconstructed flap.

**Figure 2:**
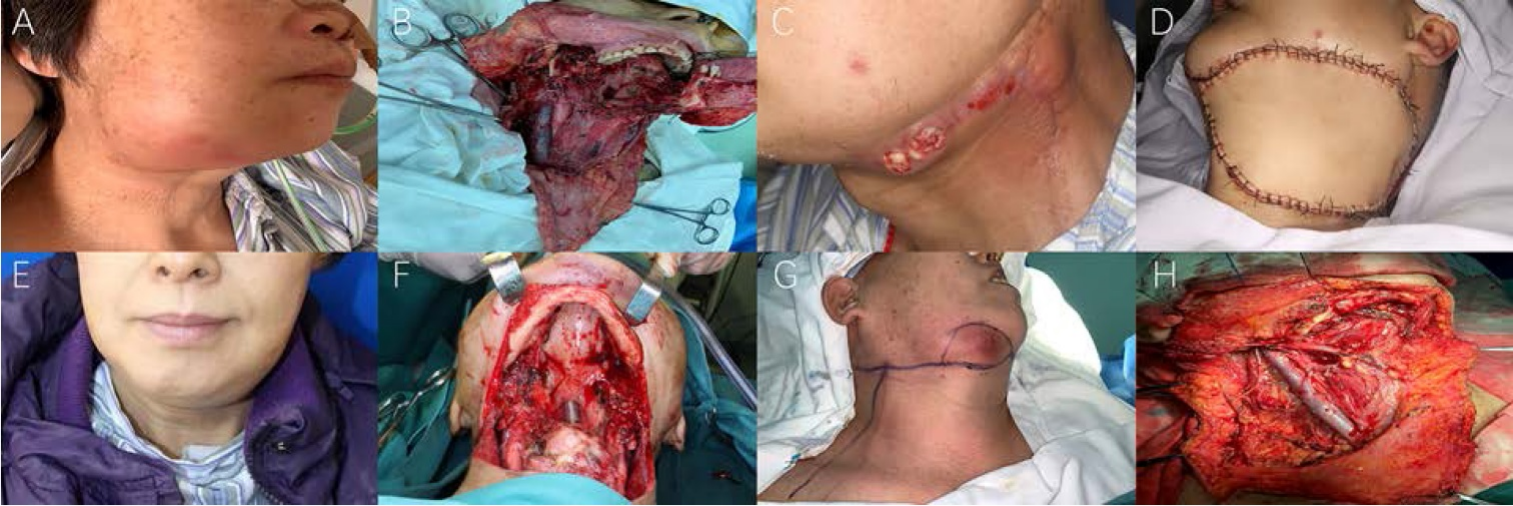
** Representative photos of oral cancer patients with bilateral ENE nodes (High resolution image is in the Supplementary material). A.** RL group: pre-op photo of Case 7 (recurrent tongue cancer) with obvious recurrent level Ib maENE and contralateral miENE metastases. **B.** RL group: intra-op photo of Case 7 (recurrent tongue cancer) after radical neck dissection of bilateral cervical nodes and near total glossectomy. **C.** RL group: pre-op photo of Case 8 (recurrent floor of mouth cancer) with obvious recurrent level Ib maENE (extensive cutaneous and carotid invasion) and contralateral miENE metastases. **D.** RL group: intra-op photo of Case 8 (recurrent floor of mouth cancer) after neck dissections and flap reconstruction. **E.** PL group: pre-op photo of Case 9 (primary tongue cancer) with bilateral maENE metastases. **F.** PL group: intra-op photo of Case 9 (primary tongue cancer) after en-bloc approach of bilateral neck dissections and total glossectomy. **G.** INM group: pre-op photo of Case 10 (prior buccal cancer with current INM) with right-sided maENE (invading mandible and skin) and left-sided miENE nodes. **H.** INM group: intra-op photo of Case 10 (prior buccal cancer with current INM) after neck dissections with right-sided marginal mandibulectomy.

On the other hand, as the focus of this study, the clinicopathologic features of bilateral ENE nodes were given considerable attention, as shown in ***Table 3***. Firstly, regarding the preoperative examination of nodes, ENE signs were not confirmed by radiologic imaging in 36 (28.1%) patients. The intraoperative findings further revealed ipsilateral metastatic lymph node fusion (or agglomeration) in approximately one-fourth (n=37, 28.9%) of the included patients. The postoperative pathological review showed that ENEma was found only in 108 (84.4%) patients, indicating the severe cervical extension (bilateral ENE group) into the surrounding tissues. A closer inspection of the pathologic reports showed obvious soft tissue involvement, rather than simply ENE presence, in the majority of patients (n=79, 61.7%). Surprisingly, mandibular involvement of ENE nodes (bilateral ENE group) was also confirmed in 26 (20.3%) patients, while hyoid involvement was noted in 9 (7.0%), showing the aggressiveness of ENE nodes. Apart from these findings, postoperative pathologic evaluations revealed that the average number of ipsilateral metastatic nodes equaled 5.3, while that on the contralateral sides equaled 4.1. For the nodes with ENE features, pathologic evidence showed an approximate average number of 2 for both sides. In addition, the presence of multilevel ENE nodes was found in these patients, with levels I-III (89, 69.5%) being the most likely sites of bilateral involvement. Fused (inseparable) dumbbell-like metastatic nodes were also reported in 37 (28.9%) patients, indicative of the seriousness of these ENE metastases. The average lymph node ratio (LNR) between metastatic nodes and all excised nodes reached 0.23±0.15, while the average ratio between ENE nodes and all excised nodes was 0.12±0.09 (***Figures 1 and 2****, Representative bilateral ENE cases and images*).

### Follow-up and Univariate survival analyses

For the unilateral ENE group, the mean follow-up time reached 33.8 months. The most frequently encountered treatment failure was locoregional recurrences (n=94, 18.8%). Eight (1.6%) cases were died to non-oncologic causes (***Supplementary Table 4***).

On the other hand, the mean follow-up for the bilateral ENE group reached only 23.2 months (TTR: 7.9 months). Most of the deaths in this group were due to failure of either locoregional control (38, 45.8%), distant metastases (20, 24.1%), or both (21, 25.3%) (***Table 4***). Either ENE-related cervical recurrence or distant metastasis was found to contribute to treatment failure in 64 (50.0%) patients.

For the unilateral ENE group, the survival analyses revealed that patients with PL enjoyed the best DFS outcome when compared with those with RL or INM status (p<0.001). However, the sides (ipsilateral/contralateral) of ENE nodes did not sway the DFS in these patients (p=0.252). The similar trend was also observed when taking TTR as the endpoint event. All these data were shown in ***Supplementary Table 5 & 6***. In the univariate analyses of all the possible demographic and oral lesion variables for these OCSCC patients with bilateral ENE nodes (***Table 5 & 6***), the treatment group (i.e., PL, RL and INM groups) was found to be significantly related to DFS (p=0.012). Positive surgical margin status also predicted a worse treatment outcome in both the whole series (p=0.002) and the RL cohort (0.020). In addition, a parallel survival impact (p=0.001) was also found: as the treatment regimens escalated (radiochemotherapy and radiochemotherapy plus anti-EGFR therapies), the DFS rate increased accordingly. However, the occurrence of perioperative complications (p=0.017) adversely affected the outcome based on our univariate analyses. The similar results were observed when taking TTR as the endpoint (***Supplementary Table 7***).

When taking the lymph nodes information into consideration (***Table 7 & Supplementary Table 8***), a number of factors were explored for their potential in revealing the treatment outcomes. Firstly, DFS was adversely affected by the maximum size of metastatic ENE nodes (p<0.001). Second, ICCR subclassification (p=0.004) and arterial nodal encasement (p=0.026) were significantly related to a worse DFS despite aggressive treatment. Unexpectedly, LNR (p=0.696) and ENE node number ratio (p=0.123) were not able to further stratify patients with bilateral ENE nodes. Besides, HPV status was not significantly associated with DFS in neither unilateral (p=0.066) or bilateral ENE groups (p=0.876).

### Comparisons between unilateral (ipsilateral/contralateral) and bilateral ENE groups

Though the distribution of treatment subgroups (PL, RL and INM) was statistically equal (p=0.660), DFS time between unilateral and bilateral ENE groups varied greatly (DFS: p<0.001), signaling the doubled power of ENE nodes towards eventual treatment failure (***Table 8***). Most other variables, such as T classifications, DOI, number of ENE nodes, muscular invasion, were largely different between unilateral and bilateral ENE groups. Surprisingly, there were insignificant differences between ENE sizes (p=0.800) and LNR (p=0.337) in these two groups.

### Multivariate Cox regression analysis for bilateral ENE group

All parameters included in the univariate analysis were further assessed using Cox multivariate regression analysis (***Table 5-7***). After adjusting for different covariables, treatment group (p=0.017), surgical margin (p=0.003), postoperative adjuvant therapy (p=0.014) and perioperative complications (p=0.036) remained independently associated with the final treatment outcome. In addition, a posterior (latter) oral subsite (p=0.037), a higher T classification (p=0.026) and skull base involvement (p=0.040) conferred the worst DFS rate in the Cox analysis. Interestingly, alongside the proven effects of the maximum size of ENE nodes (p=0.039) and arterial encasement (p=0.025), ICCR subclassification (p=0.036) was also found to adversely affect the DFS results after allowance for potential confounders. The results for unilateral ENE group could also be found in ***Supplementary Table 5***.

### Correlation between bilateral ENE nodes and other related variables

Based on the correlation analysis (***Table 9***), the number of ipsilateral lymph nodes, location of the ipsilateral ENE, fusion of ipsilateral metastatic lymph nodes and LNR showed possible correlations with the number of ipsilateral ENE (p<0.001). For the contralateral side, the number of contralateral metastatic lymph nodes, level of contralateral ENE nodes and LNR (p<0.001) were strongly correlated with the number of contralateral ENE nodes. In addition, lower levels of ipsilateral ENE nodes were possibly related to the male sex (p=0.001), a deeper DOI (p=0.038) and higher T classification (p=0.029), while lower levels of contralateral ENE nodes were frequently found in those with bone destruction (p=0.015), a higher T classification (p=0.038), fusion (blurred) of the oral lesion and cervical metastasis (p=0.024) and internal jugular obstruction due to cancer embolism (p=0.005). Moreover, the maximum size of ENE nodes was also found to be correlated with the ICCR subclassification (p<0.001).

## Discussion

Due to its significance in treatment considerations, ENE, as a discrete adverse entity, has been added to the recent AJCC classification for upgrading the nodal status of advanced OCSCCs. Specifically, ENE (with even microscopic presence) in a single node, regardless of size, will directly categorize patients into stage IV 11, 22. However, there have been concerns about ENE as a single factor for staging, disregarding other MLN information, as patients with such ENE features have diverse clinicopathologic backgrounds. ENE features can be found in metastatic nodes of different sizes and different levels of invasion and in OCSCC patients with either primary or recurrent lesions 24. Although patients with ENE nodes would most likely receive augmented treatment regimens, the outcomes available in the literature were vastly different, showing varying treatment benefits among OCSCC patients 25-27. As was shown in our study, those with unilateral ENE nodes enjoyed much better DFS results than those with bilateral ones. It was also reasonable to assume that patients with multiple ENE nodes (or a higher ENE nodal density) will have a further reduced OS rate, as reflected in our study (35.2%). This hypothesis for the specific extent of ENE concerns has also been validated, as patients with ENEma tended to receive less benefit, with a mere DFS rate of 28.7% in our results. When further stratified by admission status, the results of the INM group (patients with both INM and bilateral ENE nodes) fell largely short of expectations, with a surprising drop in the DFS rate to merely 25%, illustrating the lower therapeutic efficiency among even patients without oral lesion recurrences. We also found that the presence of bilateral ENE nodes was not a single event but was instead coupled with other important clinical parameters, especially in patients with adverse locoregional factors (T classification (p=0.026) and surgical margins (p=0.003)). Collectively, these factors conferred the worst survival probabilities. On the other hand, among nodal characteristics, the maximum size of ENE nodes (p=0.039), ICCR subclassification (p=0.036) and carotid arterial encasement (p=0.025) were found to be associated with much worse outcomes. These clinicopathologic features in patients with bilateral ENEs, especially those regarding the ENE status, were largely different from those with unilateral ENE nodes, according to the comparisons (***Table 8***).

**Figure 3:**
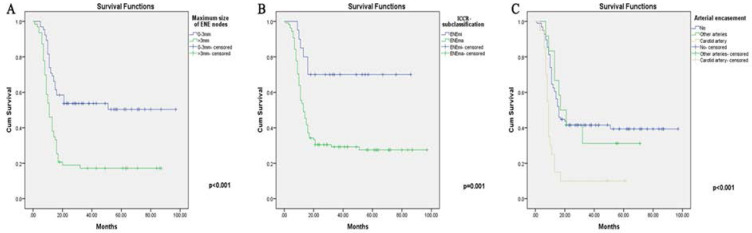
** The Kaplan-Meier curves for ENE-related variables (significant in Cox analysis) (High resolution image is in the Supplementary material). A.** Maximum size of ENE nodes. **B.** ICCR subclassification (maENE or miENE). **C.** Cervical arterial encasement.

In addition, bilateral ENE features related to general soft tissue involvement (p=0.039), together with detailed muscle invasion, arterial encasement and jugular venous embolism (obstruction), foreshadowed undesirable outcomes among OCSCC patients in the PL groups, as any major invasion into either of these structures would increase the likelihood of recurrence or distant metastasis despite aggressive locoregional treatment. The survival benefits of complete excision (negative surgical margin) and adjuvant therapies were also demonstrated in our study as proof of a standardized treatment for select cases with such nodal features. Therefore, we added our new evaluation and treatment considerations to further subcategorize and weigh the benefits of bilateral ENE features in OCSCC patients (***Figure 3***). The focus of treatment recommendations was mainly based on DFS influences of specific clinicopathologic features.

Cervical ENE (extracapsular-spread) nodes were first reported in 1974 in a treatment failure analyses of patients with OCSCC 32. Since then, the significance of ENE has been gradually recognized in head and neck cancers, mostly OCSCC and oropharyngeal cancers. Within the existing literature, the presence of ENE in MLNs was mostly reported to be associated with an increased likelihood of locoregional recurrence and distant metastasis 26, ultimately leading to lower survival rates among OCSCC patients. This was also consistent with our results. Nevertheless, the impact of ENE was quite subtle, as marked discrepancies in survival rates for OCSCC patients could be discerned between different studies, ranging from 66.6% to 38.9% 17-19. Some even asserted that more than 2 ENE nodes would confer unfavorable prognoses, while single ENE nodes did not affect overall survival 21. Others considered that the unfavorable distributions (lower levels) of multiple ENE nodes would constitute risk factors 27, 33. However, the MLN burden with bilateral ENE features might have strong relations to an inferior DFS, as demonstrated in our study. Such relations, as far as we are concerned, were proven by the lower regional control rate and the much higher possibility of distant metastasis, as reflected in Table 1. For OCSCC patients with bilateral ENE MLNs, ENEma (>2 mm) was a common feature, which was found in almost 90% of our bilateral ENE cases. In contrast with Tirelli's report 34, the ICCR subclassification of ENEmi and ENEma was found to be significantly associated with DFS in our study. In addition, the prognostic influence of other ENE features, especially multiple bilateral ENE MLNs, in RL and INM patients was rather elusive since most studies have only focused on primary OCSCC patients. Theoretically speaking, when treating recurrent OCSCC patients with multiple ENE MLNs, most surgeons would be reluctant to offer salvage surgical treatment due to the generally unfavorable DFS (15.4%), which was also confirmed in our study. We also found that age, sex or comorbidities did not significantly affect the prognosis of these OCSCC patients. In addition, patients with larger-sized (≥4 cm) oral RLs and bilateral ENE MLNs, which might entail larger flap reconstructions and intensified adjuvant therapies, should be considered for palliative modalities due to surgery-relevant treatment toxicity, as few DFS benefits and complications (p=0.036) were noted in our study. In other words, salvage treatment could be offered to select RL patients with low oral disease burdens and bilateral ENE MLNs.

It is plausible that OCSCCs in different oral subsites grow, invade the surrounding organs, and metastasize to regional lymph nodes in different ways 35-36. In our study, the retromolar trigone 4, 3.1%), lower gingiva (10, 7.8%) and bucca (8, 6.3%) were found to be associated with much lower DFS. In contrast with expectations, DOI (p=0.779), unlike T classification, was not found to be significantly related to survival outcomes 29. Surprisingly, most of the clinicopathological characteristics, such as imaging features, PNI, and oral-cervical nodal fusion, did not reach statistical significance. Unlike oral lesions, we found that the most influential factors for patients with bilateral ENE nodes were still nodal size and extent of infiltration (ICCR subclassification), implying dual considerations for extracapsular invasion and size in terms of the treatment prognosis. According to our study, the cutoff value of the maximum ENE nodes reached 3 cm (Supplementary figure 1 for receiver operating characteristic (ROC) curve cutoff value). Based on this finding, we contend that, for cases with bilateral smaller ENE nodes (<3 cm), salvage surgical treatment in combination with adjuvant treatment is still feasible, while caution should be taken for patients with larger ENE nodes (≥3 cm). In addition, OCSCC patients were mostly salvageable when both smaller ENE sizes and ENEmi were found in the postoperative pathological reports. In addition to these features, for those with ENE nodal carotid arterial encasement, most salvage surgeries might not achieve the goal of potential rescue, as approximately 90% of the cases would not gain any DFS benefit despite aggressive treatment. Interestingly, unlike the ICCR subclassification, nodal soft tissue involvement as a whole was not associated with DFS due to the influence of other covariates, while muscle invasion was found to be correlated with OS (p=0.008) in the PL group, within which direct SCM invasion largely reduced the DFS rate to approximately 25%.

Patients with ENE MLNs are always considered candidates for postoperative adjuvant therapies due to the higher risk of treatment failure 34. The striking advantage of postoperative concurrent chemoradiotherapy (CCRT) in MLN head and neck cancers was found in a collaborative analysis of two randomized phase III trials conducted in Europe by European Organization Research and Treatment of Cancer (EORTC) 22931 and in the United States by Radiation Therapy Oncology Group (RTOG) 9501^37^-^38^. However, there were controversial results regarding the role of CCRT in ENE patients, even in the aforementioned RTOG 9501 trial, as patients with ENE nodes failed to have improved long-term outcomes with the addition of cisplatin-based chemotherapy (due to a high rate of recurrence) 39. Our study suggested that patients with bilateral ENE MLNs could gain additional survival benefit when the adjuvant regimens were escalated. In addition, patients receiving targeted therapies such as EGFR-inhibited drugs in combined treatment approaches with CCRT tend to have slightly better outcomes (50% vs. 46.2%). However, it should also be noted that toxicity increases with such additions; patients receiving these protocols were mostly of a younger age or had fewer comorbidities.

**Figure 4:**
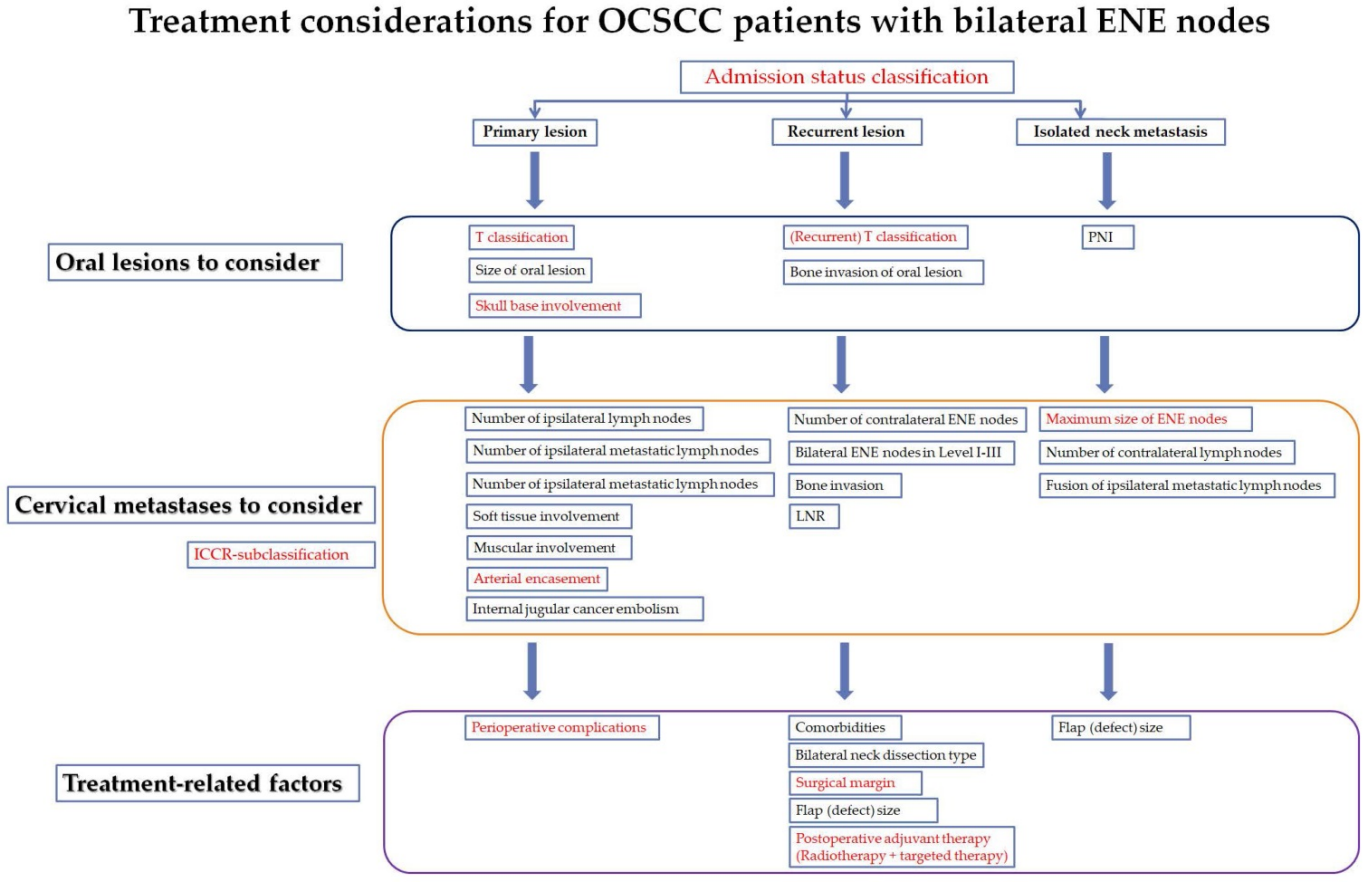
** The proposed treatment considerations and risk factors for oral cancer patients with bilateral ENE nodes (under different PL, RL and INM clinical situations) (High resolution image is in the Supplementary material).** Red fonts: The variables also significant in the multivariate Cox analysis for the whole series of patients.

The current study had some limitations due to its retrospective design, including the lack of a randomized patient population and associated biases. Due to the low incidence of bilateral ENE MLNs in OCSCC patients, we tended to increase the study population by enrolling patients with different prior treatment histories, which might have partially influenced the general analysis of the data. The trend in our study that increased (escalated) treatment regimens led to better survival should be viewed with caution as these were mostly prescribed to younger patients with less comorbidities. The result could not be directly extended to those of older ages or with more comorbidities. Thus, further multicenter studies are needed to investigate whether OCSCC patients with bilateral ENE features should be considered for different subgroups and treatment considerations (***Figure 4***).

## Conclusion

Bilateral cervical metastases with ENE features, though uncommon, represent serious regional burdens and lead to lower-than-expected treatment outcomes, especially in those with RLs or INMs. A fairly large number of OCSCC patients with advanced oral lesions gain little benefit from intensified salvage surgical treatment. Such treatment should instead be offered to select salvageable patients with smaller bilateral ENE nodes (<3 cm), lower ENE subclassifications and no arterial nodal encasement.

## Supplementary Material

Supplementary tables.

## Figures and Tables

**Table 1:** Demographics and treatment summary for patients with bilateral ENE nodes

Variables	The whole groups		The PL group		The RL group		The INM group	
	N (%)	The DFS rate	N (%)	The DFS rate	N (%)	The DFS rate	N (%)	The DFS rate
**Treatment group**								
Primary lesions* (PL)	85 (66.4)	42.4						
Recurrent lesions (RL)	26 (20.3)	15.4						
Isolated neck metastases (INM)	17 (13.3)	29.4						
**Age**								
31-59	57 (44.5)	42.1	39 (45.9)	43.6	12 (46.2)	33.3	6 (35.3)	50.0
60-87	71 (55.5)	29.6	46 (54.1)	41.3	14 (53.8)	0.0	11 (64.7)	18.2
Sex								
Male	97 (75.8)	36.1	72 (84.7)	43.1	15 (57.7)	6.7	10 (58.8)	30.0
Female	31 (24.3)	32.3	13 (15.3)	38.5	11 (42.3)	27.3	7 (41.2)	28.6
**Histories of smoking and alcohol**								
Yes	59 (46.1)	40.7	42 (49.4)	52.4	12 (46.2)	8.3	5 (29.4)	20.0
No	69 (53.9)	30.4	43 (50.6)	32.6	14 (53.8)	21.4	12 (70.6)	33.3
**Comorbidities**								
Yes	54 (42.2)	38.9	38 (44.7)	47.4	9 (34.6)	0.0	7 (41.2)	42.9
No	74 (57.8)	32.4	47 (55.3)	38.3	17 (65.4)	23.5	10 (58.8)	20.0
**History of prior treatment**								
Surgery with/without adjuvant therapy	43 (33.6)	20.9	0		26 (100.0)	15.4	17 (100.0)	29.4
Adjuvant therapy alone	33 (25.8)	36.4	33 (38.8)	36.4	0		0	
None	52 (40.6)	46.2	52 (61.2)	46.2	0		0	
**Ipsilateral neck dissection^a^**								
SOND	17 (13.3)	35.3	9 (10.6)	55.6	5 (19.2)	0.0	3 (17.6)	33.3
Extended SOND	20 (15.6)	40.0	14 (16.5)	42.9	6 (23.1)	33.3		
RND	91 (71.1)	34.1	62 (72.9)	40.3	15 (57.7)	13.3	14 (82.4)	28.6
**Contralateral neck dissection**								
SOND	34 (26.6)	32.4	19 (22.4)	42.1	10 (38.5)	20.0	5 (29.4)	20.0
Extended SOND	28 (21.9)	42.9	20 (23.5)	45.0	5 (19.2)	20.0	3 (17.6)	66.7
RND	66 (51.6)	33.3	46 (54.1)	41.3	11 (42.3)	9.1	9 (52.9)	22.2
**En-bloc resection**								
Yes	73 (57.0)	38.4	55 (64.7)	45.5	18 (69.2)	16.7	0	
No	55 (43.0)	30.9	30 (35.3)	36.7	8 (30.8)	12.5	17 (100.0)	29.4
**Surgical margin**								
Positive	12 (9.4)	8.3	5 (5.9)	20.0	5 (19.2)	0.0	2 (22.8)	0.0
Negative	116 (90.6)	37.9	80 (94.1)	43.8	21 (80.8)	19.0	15 (77.2)	29.4
**Size of flap (Length of skin island)**								
No	18 (14.1)	44.4	8 (9.4)	37.5	2 (7.7)	0.0	8 (47.1)	62.5
0-10cm	18 (14.1)	50.0	13 (15.3)	61.5	2 (7.7)	50.0	3 (17.6)	0.0
10-15cm	49 (38.3)	32.7	36 (42.4)	38.9	8 (30.8)	25.0	5 (29.4)	0.0
15-20cm	24 (18.8)	25.0	17 (20.0)	35.3	7 (26.9)	0.0	0	
≥20cm	19 (14.8)	31.6	11 (12.9)	45.5	7 (26.9)	14.3	1 (5.9)	0.0
**Flap type**								
Anterolateral thigh flap	61 (47.7)	32.8	44 (51.8)	43.2	14 (53.8)	7.1	3 (17.6)	0.0
Fibular flap	2 (1.6)	50.0	2 (2.4)	50.0	0		0	
Radial forearm flap	10 (7.8)	40.0	9 (10.6)	44.4	0		1 (5.9)	0.0
Latissimus dorsi flap	3 (2.3)	33.3	1 (1.2)	0.0	2 (7.7)	50.0	0	
Pectoralis myocutaneous flap	31 (24.2)	35.5	19 (22.4)	47.4	7 (26.9)	28.6	5 (29.4)	0.0
Direct close or regional flap	21 (16.4)	38.1	10 (17.8)	30.0	3 (11.5)	0.0	8 (47.1)	62.5
**Perioperative complications^&^**								
Surgical site infection	12 (9.4)	16.7	8 (9.4)	25.0	3 (11.5)	0.0	1 (5.9)	0.0
Pulmonary infection	32 (25.0)	15.6	22 (25.9)	22.7	7 (26.9)	0.0	3 (17.6)	0.0
Chyle leakage	4 (3.1)	25.0	1 (1.2)	100.0	2 (7.7)	0.0	1 (5.9)	0.0
Orocutaneous fistula	8 (6.3)	0.0	6 (7.1)	0.0	2 (7.7)	0.0	0	
Flap necrosis	6 (4.7)	16.7	5 (5.9)	0.0	1 (3.8)	100.0	0	
Hematoma	2 (1.6)	0.0	2 (2.4)	0.0	0		0	
Delirium	3 (2.3)	33.3	2 (2.4)	50.0	1 (3.8)	0.0	0	
Wound dehiscence	10 (7.8)	30.0	5 (5.9)	20.0	5 (19.2)	40.0	0	
Deep venous thrombosis	4 (3.1)	0.0	4 (4.7)	0.0	0		0	
**HPV status**								
Yes	3 (2.3)	33.3	2 (2.4)	50.0	0		1 (5.9)	0.0
No	20 (15.6)	30.0	14 (16.5)	35.7	5 (19.2)	0.0	1 (5.9)	100.0
Unknown	105 (82.0)	36.2	69 (81.2)	43.5	21 (80.8)	19.0	15 (88.2)	26.7
**Postoperative adjuvant therapy^#^**								
Radiotherapy	31 (24.2)	22.6	17 (20.0)	25.0	8 (30.8)	0.0	6 (35.3)	33.3
Chemotherapy	3 (2.3)	33.3	1 (1.2)	0.0	2 (7.7)	50.0	0	
Radio-chemotherapy	52 (40.6)	46.2	40 (47.1)	55.0	8 (30.8)	12.5	4 (23.5)	25.0
Radiotherapy and anti-EGFR therapy	15 (11.7)	26.7	12 (14.1)	25.0	1 (3.8)	100.0	2 (11.8)	0.0
Chemotherapy and anti-EGFR therapy	3 (2.3)	33.3	0		3 (11.5)	33.3	0	
Radio-chemotherapy and anti-EGFR therapy	12 (9.4)	50.0	9 (10.6)	55.6	1 (3.8)	0.0	2 (11.8)	100.0
None	12 (9.4)	8.3	6 (7.1)	16.7	3 (11.5)	0.0	3 (17.6)	0.0

NA: Not Applicable; *: Including a case with synchronous primary lesions in the tongue and thyroid; ^a^: SOND: Supra-omohyoid neck dissection (Level I-III); extended SOND: extended supra-omohyoid neck dissection (Level I-IV); RND: Radical neck dissection (Level I-V); ^#^: Ten cases in the RL group had received radiotherapy before, and were treated with re-radiotherapy after surgery. The overall survival of them was 20.0%; &: Some cases had multiple complications.

**Table 2:** The characteristics of primary or recurrent oral lesions in patients with bilateral ENE nodes

Variables	The whole groups		The PL group		The RL group		The INM group	
	N (%)	The DFS rate	N (%)	The DFS rate	N (%)	The DFS rate	N (%)	The DFS rate
**Primary or recurrent subsite**								
Tongue	60 (46.9)	46.7	40 (47.1)	55.0	11 (42.3)	27.3	9 (52.9)	33.3
Floor of mouth	34 (26.6)	38.2	24 (28.2)	45.8	9 (34.6)	11.1	1 (5.9)	100.0
Buccal mucosa	8 (6.3)	12.5	2 (2.4)	0.0	3 (11.5)	0.0	3 (17.6)	33.3
Lower Gingiva	10 (7.8)	10.0	7 (8.2)	14.3	2 (7.7)	0.0	1 (5.9)	0.0
Retromolar trigone	4 (3.1)	0.0	3 (3.5)	0.0	1 (3.8)	0.0	0	0.0
Hard palate	6 (4.7)	16.7	6 (7.1)	16.7	0	0.0	0	0.0
Upper Gingiva	6 (4.7)	16.7	3 (3.5)	33.3	0	0.0	3 (17.6)	0.0
**Pathologic grade**								
I	2 (1.6)	0.0	1 (1.2)	0.0	0	0.0	1 (5.9)	0.0
II	77 (60.2)	40.3	54 (63.5)	48.1	18 (69.2)	11.1	5 (29.4)	60.0
III	49 (38.3)	28.6	30 (35.3)	33.3	8 (30.8)	25.0	11 (64.7)	18.2
T classification*								
T2	11 (8.6)	54.5	2 (2.4)	100.0	0	NA	9 (52.9)	44.4
T3	63 (49.2)	44.4	50 (58.8)	48.0	9 (34.6)	33.3	4 (23.5)	25.0
T4	54 (42.2)	20.4	33 (38.8)	30.3	17 (65.4)	5.9	4 (23.5)	0.0
**Size of oral lesion**								
0-2 cm	5 (3.9)	40.0	0	0.0	0	0.0	5 (29.4)	40.0
2-4 cm	40 (31.3)	52.5	25 (29.4)	64.0	5 (19.2)	40.0	10 (58.8)	30.0
4-6 cm	57 (44.5)	35.1	45 (52.9)	44.4	9 (34.6)	0.0	3 (17.6)	0.0
>6 cm	35 (27.3)	25.9	22 (25.9)	18.2	13 (50.0)	15.4	0	NA
**DOI>10 mm**								
Yes	115 (89.8)	34.8	80 (94.1)	42.5	26 (100.0)	15.4	9 (52.9)	22.2
No	13 (10.2)	38.5	5 (5.9)	40.0	0	0.0	8 (47.1)	37.5
**Midline involvement**								
Yes	96 (75.0)	38.5	65 (76.5)	47.7	22 (84.6)	13.6	9 (52.9)	33.3
No	32 (25.0)	25.0	20 (23.5)	25.0	4 (15.4)	25.0	8 (47.1)	25.0
**PNI**								
Yes	69 (53.9)	31.9	45 (52.9)	44.4	16 (61.5)	6.3	8 (47.1)	12.5
No	59 (46.1)	39.0	40 (47.1)	40.0	10 (38.5)	30.0	9 (52.9)	44.4
**Bone destruction (oral lesion)**								
Yes	48 (37.5)	20.8	28 (32.9)	32.1	16 (61.5)	6.3	4 (23.5)	0.0
No	80 (62.5)	43.8	57 (67.1)	47.4	10 (38.5)	30.0	13 (76.5)	38.5
**Skull base involvement**								
Yes	8 (6.3)	37.5	8 (9.4)	37.5	0	0.0	0	0.0
No	120 (93.8)	35.0	77 (90.6)	42.9	26 (100.0)	15.4	17 (100.0)	29.4

NA: Not Applicable; *: The T classification of the RL or INM group was based on the pathological characteristics of their prior primary lesions according to AJCC system.

**Table 3:** The characteristics of metastatic lymph nodes and ENE features in patients with bilateral ENE nodes

Variables	The whole groups		The PL group		The RL group		The INM group	
	N (%)	The DFS rate	N (%)	The DFS rate	N (%)	The DFS rate	N (%)	The DFS rate
**ENE found via preoperative imaging**								
Bilateral ENE	55 (43.0)	29.1	30 (35.3)	43.3	15 (57.7)	6.7	10 (58.8)	20.0
Ipsilateral ENE	26 (20.3)	34.6	20 (23.5)	40.0	3 (11.5)	33.3	3 (17.6)	33.3
Contralateral ENE	11 (8.6)	27.3	7 (8.2)	28.6	3 (11.5)	33.3	1 (5.9)	0.0
No	36 (28.1)	47.2	28 (32.9)	46.4	5 (19.2)	40.0	3 (17.6)	66.7
**ICCR-subclassification**								
ENEma	108 (84.4)	28.7	69 (81.2)	34.8	24(92.3)	16.7	15(88,2)	20.0
ENEmi	20 (15.6)	70.0	16 (18.8)	75.0	2(7.7)	0.0	2(11.8)	100.0
**Fusion of oral lesion and metastatic lymph node**								
Yes	32 (25.0)	25.0	22 (25.9)	27.3	9(34.6)	22.2	1(5.9)	0.0
No	96 (75.0)	38.5	63 (74.1)	47.6	17(65.4)	11.8	16(94.1)	31.3
**Maximum size of ENE nodes**								
Mean ± standard deviation	3.0±0.9	NA	2.8±0.7	NA	2.8±0.4	NA	3.9±1.5	NA
**Number of ipsilateral lymph nodes**								
Mean ± standard deviation	23.1±10.2	NA	24.5±9.8	NA	20.0±10.6	NA	20.8±10.5	NA
**Number of contralateral lymph nodes**								
Mean ± standard deviation	20.8±10.6	NA	21.7±10.4	NA	17.9±9.2	NA	20.7±13.4	NA
**Number of ipsilateral metastatic lymph nodes**								
Mean ± standard deviation	5.3±3.9	NA	5.7±4.1	NA	4.5±3.6	NA	4.6±3.6	NA
**Number of contralateral metastatic lymph nodes**								
Mean ± standard deviation	4.1±5.3	NA	3.8±3.5	NA	4.3±3.4	NA	5.7±11.8	NA
**Number of ipsilateral ENE nodes**								
Mean ± standard deviation	2.6±2.1	NA	2.7±2.0	NA	2.6±2.6	NA	2.4±1.6	NA
**Number of contralateral ENE nodes**								
Mean ± standard deviation	2.0±2.3	NA	1.7±1.3	NA	2.3±1.6	NA	3.1±5.2	NA
**Level of ipsilateral ENE nodes**								
I-III	101 (78.9)	35.6	68 (80.0)	45.6	20 (76.9)	5.0	13 (76.5)	30.8
IV-V	27 (21.1)	33.3	17 (20.0)	29.4	6 (23.1)	50.0	4(23.5)	25.0
**Level of contralateral ENE nodes**								
I-III	110 (85.9)	34.5	77 (90.6)	40.3	19(73.1)	15.8	14(82.4)	28.6
IV-V	18 (14.1)	38.9	8 (9.4)	62.5	7(26.9)	14.3	3(21.4)	33.3
**Bilateral ENE nodes in I-III level**								
Yes	89 (69.5)	36.0	62 (72.9)	45.2	14(53.8)	0.0	13(76.5)	30.8
No	39 (30.5)	33.3	23 (27.1)	34.8	12(46.2)	30.0	4(23.5)	25.0
**Fusion of ipsilateral metastatic lymph nodes**								
Yes	37 (28.9)	29.7	23 (27.1)	34.8	8(30.8)	12.5	6(35.3)	33.3
No	91 (71.1)	37.4	62 (72.9)	45.2	18(69.2)	16.7	11(64.7)	18.2
**Fusion of contralateral metastatic lymph nodes**								
Yes	25(19.5)	28.0	13(15.3)	38.5	7(26.9)	14.3	5(29.4)	20.0
No	103(80.5)	36.9	72(84.7)	43.1	19(73.1)	15.8	12(70.6)	33.3
**Soft tissue involvement**								
Yes	79(61.7)	25.3	46(54.1)	41.3	19(73.1)	21.1	14(82.4)	14.3
No	49(38.3)	51.0	39(45.9)	43.6	7(26.9)	0.0	3(17.6)	100.0
**Muscular invasion**								
Sternocleidomastoid muscle (SCM)	64(50.0)	23.4	37(43.5)	24.3	16(61.5)	25.0	11(64.7)	18.2
Other muscles	14(10.9)	35.7	9(10.6)	55.6	3(11.5)	0.0	2(15.4)	0.0
No	50(39.1)	50.0	39(45.9)	56.4	7(26.9)	0.0	4(23.5)	75.0
**Arterial encasement**								
Carotid artery	20(15.6)	10.0	11(12.9)	18.2	4(15.4)	0.0	6(35.3)	0.0
Other arteries	12(9.4)	33.3	8(9.4)	50.0	3(11.5)	0.0	0	NA
No	96(75)	40.6	66(77.6)	45.5	19(73.1)	21.1	11(64.7)	45.5
**Internal jugular cancer embolism**								
Yes	14(10.9)	21.4	8(9.4)	37.5	4(15.4)	0.0	2(15.4)	0.0
No	114(89.1)	36.8	77(90.6)	42.9	22(84.6)	18.2	15(88.2)	33.3
**Lymph node necrosis**								
Yes	43(33.6)	37.2	25(29.4)	44.0	10(38.5)	30.0	8(47.1)	25.0
No	85(66.4)	34.5	60(70.6)	41.7	16(61.5)	6.3	9(52.9)	33.3
**Bone involvement**								
Mandible	26(20.3)	26.9	11(12.9)	36.4	7(26.9)	28.6	8(47.1)	12.5
Hyoid	9(7.0)	11.1	6(7.1)	16.7	2(7.7)	0.0	1(5.9)	0.0
No	93(72.7)	40.0	68(80.0)	45.6	17(65.4)	11.8	8(47.1)	50.0
Lymph node ratio (LNR)*								
Mean ± standard deviation	0.23±0.15	NA	0.22±0.14	NA	0.26±0.14	NA	0.24±0.17	NA
Number ratio between ENE and excised nodes^&^								
Mean ± standard deviation	0.12±0.09	NA	0.11±0.08	NA	0.15±0.10	NA	0.15±0.12	NA

NA: Not Applicable; *: The number of metastatic nodes divided by the total number of excised nodes; &: The number of ENE nodes divided by the total number of excised nodes.

**Table 4:** Summary of death causes of patients with bilateral ENE nodes

Variables (N, %)	N (%)			
	The whole groups	The PL group	The RL group	The INM group
**Death causes (Overall)**				
Locoregional recurrence	38 (45.8)	23 (46.9)	11 (50.0)	4 (33.3)
Distant metastasis	20 (24.1)	10 (20.4)	7 (31.8)	3 (25.0)
Distant metastasis and locoregional recurrence	21 (25.3)	14 (28.6)	3 (13.6)	4 (33.3)
Complication-related cause	2 (2.4)	0	1 (4.5)	1 (8.3)
Non-oncologic cause	2 (2.4)	2 (4.1)	0	0
**Death causes (limited to cervical recurrence or distant metastasis)**				
Yes	64 (50.0)	36 (42.4)	17 (65.4)	11 (64.7)
No	19 (14.8)	13 (15.3)	5 (19.2)	1 (5.9)

**Table 5:** The Univariate and Cox regression DFS survival analysis of demographics and treatment in patients with bilateral ENE nodes

Variables	The whole groups		The PL group		The RL group		The INM group	
	Univariate analysis	Multivariate analysis (OR,95%CI)	Univariate analysis	Multivariate analysis (OR,95%CI)	Univariate analysis	Multivariate analysis (OR,95%CI)	Univariate analysis	Multivariate analysis (OR,95%CI)
Treatment group	0.012	0.017(1.433,1.066~1.928)						
Age	0.166		0.595		0.282		0.370	
Sex	0.469		0.716		0.751		0.928	
Histories of smoking and alcohol	0.279		0.111		0.627		0.616	
Comorbidities	0.355		0.284		0.031	0.020(4.646,1.270~16.989)	0.369	
History of prior treatment	0.110		0.186		NA		NA	
Ipsilateral neck dissection	0.566		0.548		0.423	0.001(3.643,1.698~7.815)	0.848	
Contralateral neck dissection	0.397		0.778		0.674	0.004(2.772,1.378~5.574)	0.352	
En-bloc resection	0.090		0.190	0.055(0.561,0.311~1.011)	0.345	0.071(0.319,0.092~1.103)	NA	
Surgical margin	0.002	0.003(2.742,1.418~5.300)	0.218		0.020	<0.001(22.233,4.017~123.054)	0.419	
Flap size	0.173		0.484		0.951	0.004(0.373,0.189~0.736)	0.013	0.013(1.756,1.124~2.745)
HPV status	0.876		0.745		0.785		0.785	
Flap type	0.944		0.492		0.589		0.028	
Postoperative adjuvant therapy	0.001	0.014(0.836,0.725~0.964)	0.027		0.053	0.005(0.535,0.345~0.831)	0.800	
Perioperative complications	0.017	0.036(2.742,1.418~5.300)	0.002	0.001(2.803,1.529~5.138)	0.383		0.507	

DFS: Disease-free survival; NA: Not applicable; *: Including a case whose primary lesions were found both in the tongue and thyroid, but mostly the tongue; HPV: human papillomavirus.

**Table 6:** The Univariate and Cox regression DFS analysis of the characteristics of primary or recurrent oral lesions in patients with bilateral ENE nodes

Variables	The whole groups		The PL group		The RL group		The INM group	
	Univariate analysis	Multivariate analysis (OR,95%CI)	Univariate analysis	Multivariate analysis (OR,95%CI)	Univariate analysis	Multivariate analysis (OR,95%CI)	Univariate analysis	Multivariate analysis (OR,95%CI)
Primary or recurrent subsite	0.052	0.037	0.152		0.328		0.271	
Pathological grade	0.331		0.393		0.983		0.948	
T classification	0.012	0.026(2.158,1.098~4.244)	0.092	0.047(2.521,1.011~6.286)	0.304	0.008(80.346,3.101~2081.907)	0.018	
Size of oral lesion	0.073		0.016	0.017(1.822,1.111~2.988)	0.659		0.188	0.055(0.028,0.001~1.081)
DOI>10 mm	0.779		0.840		NA		0.118	
Midline involvement	0.295		0.107		0.545	0.096(3.744,0.790~17.752)	0.835	
PNI	0.506		0.643		0.248	0.078(2.780,0.893~8.654)	0.175	0.034(48.759,1.339~1775.527)
Bone destruction (oral lesion)	0.043		0.398	0.07(0.360,0.119~1.088)	0.508	0.035(0.026,0.001~0.769)	0.054	
Skull base involvement	0.796	0.040(0.328,0.113~0.950)	0.793	0.086(0.314,0.084~1.177)	NA		NA	

DFS: Disease-free survival; NA: Not Applicable.

**Table 7:** The Univariate and Cox regression DFS analysis of metastatic lymph nodes in patients with bilateral ENE nodes

Variables	The whole groups		The PL group		The RL group		The INM group	
	Univariate analysis	Multivariate analysis (OR,95%CI)	Univariate analysis	Multivariate analysis (OR,95%CI)	Univariate analysis	Multivariate analysis (OR,95%CI)	Univariate analysis	Multivariate analysis (OR,95%CI)
ENE found via preoperative imaging	0.075		0.840		0.296		0.121	
ICCR-subclassification	0.004	0.037(2.523,1.056~6.028)	0.013		0.405		0.295	
Fusion of oral lesion and metastatic lymph nodes	0.271		0.113		0.272		0.169	
Maximum size of ENE nodes	<0.001	0.047(1.248,1.003~1.552)	0.004		0.639		0.018	0.003(3.397,1.514~7.619)
Number of ipsilateral lymph nodes	0.530		0.621	0.021(1.062,1.009~1.119)	0.432		0.041	
Number of contralateral lymph nodes	0.589		0.952		0.279		0.458	0.039(1.054,1.003~1.109)
Number of ipsilateral metastatic lymph nodes	0.584		0.888	0.043(0.665,0.448~0.988)	0.239		0.836	
Number of contralateral metastatic lymph nodes	0.911		0.611		0.063		0.400	
Number of ipsilateral ENE nodes	0.686		0.312		0.648		0.848	
Number of contralateral ENE nodes	0.511		0.209		0.844	<0.001(2.764,1.588~4.812)	0.424	
Level of ipsilateral ENE nodes	0.914		0.411		0.050		0.632	
Level of contralateral ENE nodes	0.739		0.280		0.655		0.912	
Bilateral ENE nodes in I-III level	0.850		0.633		0.008	0.001(31.032,4.275~225.274)	0.632	
Fusion of ipsilateral metastatic lymph nodes	0.388		0.290		0.936		0.578	0.038(0.078,0.007~0.872)
Fusion of contralateral metastatic lymph nodes	0.214		0.858		0.454		0.298	
Soft tissue involvement	0.003		0.016	0.039(0.075,0.006~0.878)	0.169		0.190	
Muscular invasion	0.002		0.003	0.008(5.270,1.556~17.843)	0.116		0.168	
Arterial encasement	0.001	0.028(1.426,1.039~1.957)	0.038	0.001(3.726,1.729~8.027)	0.238		0.054	
Internal jugular cancer embolism	0.090		0.795	0.013(0.052,0.005~0.542)	0.024		0.036	
metastatic node necrosis	0.930		0.971		0.014		0.650	
Bone involvement	0.013		0.101		0.990	0.004(10.261,2.149~48.986)	0.051	
HPV	0.876		0.745		0.785		0.785	
LNR	0.696		0.957		0.155	0.001(<0.001,<0.001~0.001)	0.183	
Number ratio between ENE and excised nodes	0.123		0.914		0.779		0.235	

DFS: Disease-free survival; NA: Not Applicable.

**Table 8:** The comparisons of variable in patients with unilateral v.s. bilateral ENE nodes

Unilateral v.s. Bilateral ENE metastasis (variables)	95%CI		F	P
	Lower	Upper		
TTR time^&^	29.695	33.567	19.256	<0.001
DFS time^&^	31.826	35.41	21.776	<0.001
Treatment group	1.437	1.552	0.194	0.660
DOI>10mm	0.563	0.646	182.429	<0.001
Surgical margin	0.034	0.068	6.159	0.013
T classification*	2.977	3.087	31.435	<0.001
Maximum size of ENE nodes	3.503	3.694	0.064	0.800
ICCR-subclassification	1.765	1.828	2.214	0.137
Number of dissected lymph nodes	24.655	27.231	274.456	<0.001
Number of metastatic lymph nodes	4.334	5.192	143.845	<0.001
Number of ipsilateral ENE nodes^	1.596	1.882	38.614	<0.001
Number of contralateral ENE nodes^	0.570	0.883	76.728	<0.001
Level of ipsilateral ENE nodes^	0.974	1.058	22.063	<0.001
Level of contralateral ENE nodes^	0.342	0.433	510.668	<0.001
Bone involvement	0.108	0.162	27.361	<0.001
Internal jugular cancer embolism	0.075	0.122	0.211	0.646
Soft tissue involvement	0.455	0.534	9.807	0.002
Fusion of oral lesion and metastatic lymph node	0.036	0.072	148.841	<0.001
Arterial encasement	0.054	0.095	80.299	<0.001
Muscular invasion	0.476	0.554	5.752	0.017
metastatic node necrosis	0.243	0.313	2.669	0.103
ENE found via preoperative imaging	0.450	0.529	35.549	<0.001
LNR	0.233	0.273	0.923	0.337
Number ratio between ENE and excised nodes	0.148	0.184	5.841	0.016

DFS: Disease-free survival; TTR: Time-to-relapse; NA: Not Applicable; ^: Only patients with ipsilateral or contralateral ENE nodes were counted.

**Table 9:** The correlation analysis of bilateral ENE node presence and its relevant factors

Variables	Co-variates	p values
Number of ipsilateral ENE nodes	Number of ipsilateral metastatic nodes	<0.001
	Fusion of ipsilateral metastatic nodes	<0.001
	LNR	<0.001
	Surgical margin	0.022
	Perioperative complications	0.04
Number of contralateral ENE nodes	Treatment group	0.024
	Comorbidities	0.038
	Bone destruction (oral lesion)	0.029
	Number of contralateral lymph nodes	0.002
	Number of ipsilateral metastatic lymph nodes	0.002
	Number of contralateral metastatic lymph nodes	<0.001
	Level of contralateral ENE nodes	0.001
	Fusion of contralateral metastatic nodes	0.002
	LNR	<0.001
	T classification	0.046
Level of ipsilateral ENE nodes	Number of ipsilateral metastatic lymph nodes	<0.001
	Number of ipsilateral ENE nodes	<0.001
	LNR	0.038
	Number ratio between ENE and excised nodes	0.001
	Postoperative adjuvant therapy	0.015
Level of contralateral ENE nodes	Number of contralateral metastatic lymph nodes	<0.001
	Number of contralateral ENE nodes	<0.001
	Fusion of ipsilateral metastatic nodes	0.039
	Fusion of contralateral metastatic nodes	0.031
	Internal jugular cancer embolism	0.001
	Arterial encasement	0.012
	LNR	<0.001
	Number ratio between ENE and excised nodes	0.002
	Maximum size of ENE nodes	0.042
	Fusion of oral lesion and metastatic lymph nodes	0.012
	ENE found via preoperative imaging	0.015
Maximum size of ENE nodes	ICCR-subclassification	<0.001
	Treatment group	<0.001
	Fusion of ipsilateral metastatic nodes	<0.001
	Fusion of contralateral metastatic nodes	<0.001
	PNI	0.026
	Internal jugular cancer embolism	<0.001
	Soft tissue involvement	<0.001
	Muscular invasion	<0.001
	Arterial encasement	<0.001
	metastatic node necrosis	<0.001
	Bone involvement	<0.001
	Number ratio between ENE and excised nodes	<0.001
	Fusion of oral lesion and metastatic lymph nodes	<0.001
	ENE found via preoperative imaging	<0.001

## Abbreviations

ENE: extranodal extension
MLN: metastatic lymph node
OCSCC: oral cavity squamous cell carcinoma
HPV: human papillomavirus
EGFR: epithelial growth factor receptor
DFS: disease-free survival
TTR: time-to-relapse
AJCC: American Joint Committee on Cancer
PL: primary lesion
RL: recurrent lesion
INM: isolated neck metastasis
DOI: depth of invasion
PNI: perineural invasion
INM: isolated neck metastasis
LNR: lymph node ratio
SOND: supra-omohyoid neck dissection
RND: radical neck dissection
DVT: deep venous thrombosis
ICCR: International Collaboration of Cancer Reporting
CCRT: concurrent chemoradiotherapy
