# Supplementary material for: Extranodal Extension in Bilateral Cervical Metastases: A predictor of Undesirable Survival Outcomes despite Aggressive Salvage Treatment in Oral Cancer Patients
Source: J Cancer. 2021 Aug 3;12(19):5848–63. doi: 10.7150/jca.60152 (PMC8408102; doi:10.7150/jca.60152)
Supplement: Supplementary file 1 — Supplementary tables. [file jcav12p5848s1.pdf]

Supplementary Table 1. Demographics and treatment summary for patients with unilateral (ipsi- or contralateral) ENE nodes

| Variables | The whole groups | | The PL group | | The RL group | | The INM group | |
|---|---|---|---|---|---|---|---|---|
| | N(%) | The DFS rate | N(%) | The DFS rate | N(%) | The DFS rate | N(%) | The DFS rate |
| **Treatment group** | | | | | | | | |
| Primary lesions (PL) | 326(65.1) | 221(67.8) | | | | | | |
| Recurrent lesions (RL) | 99(19.8) | 36(36.4) | | | | | | |
| Isolated neck metastases (INM) | 76(15.2) | 33(43.4) | | | | | | |
| **Age** | | | | | | | | |
| 26-59 | 284(56.7) | 175(61.6) | 185(56.7) | 135(73.0) | 58(58.6) | 20(34.5) | 41(53.9) | 20(48.8) |
| 60-89 | 217(43.3) | 115(53.0) | 141(43.3) | 86(61.0) | 41(41.4) | 16(39.0) | 35(46.1) | 13(37.1) |
| **Sex** | | | | | | | | |
| Male | 331(66.1) | 190(57.4) | 219(67.2) | 144(65.8) | 61(61.6) | 25(41.0) | 51(67.1) | 21(41.2) |
| Female | 170(33.9) | 100(58.8) | 107(32.8) | 77(72.0) | 38(38.4) | 11(29.0) | 25(32.9) | 12(48.0) |
| **Histories of smoking and alcohol** | | | | | | | | |
| Yes | 275(54.9) | 161(58.6) | 187(57.4) | 125(66.8) | 48(48.5) | 20(41.7) | 40(52.6) | 16(40.0) |
| No | 226(45.1) | 129(57.1) | 139(42.6) | 96(69.1) | 51(51.5) | 16(31.4) | 36(47.4) | 17(47.2) |
| **Comorbidities** | | | | | | | | |
| Yes | 304(60.7) | 173(56.9) | 207(63.5) | 133(64.3) | 53(53.5) | 21(39.6) | 44(57.9) | 19(43.2) |
| No | 197(39.3) | 117(59.4) | 119(36.5) | 88(74.0) | 46(46.5) | 15(32.6) | 32(42.1) | 14(43.8) |
| **Neck dissection in the other hospital before** | | | | | | | | |
| Yes | 77(15.4) | 30(39.0) | 0 | NA | 61(61.6) | 23(37.7) | 16(21.1) | 7(43.8) |
| No | 424(84.6) | 260(61.3) | 326(100.0) | 221(67.8) | 38(38.4) | 13(34.2) | 60(78.9) | 26(43.3) |
| **History of prior treatment** | | | | | | | | |
| Surgery with/without adjuvant therapy | 169(33.7) | 65(38.5) | 0 | NA | 93(93.9) | 32(34.4) | 76(100.0) | 33(43.4) |
| Adjuvant therapy alone | 62(12.4) | 45(72.6) | 56(17.2) | 41(73.2) | 6(6.1) | 4(66.7) | 0.0000 | NA |
| None | 270(53.9) | 180(66.7) | 270(82.8) | 180(66.7) | 0 | NA | 0.0000 | NA |
| **HPV status** | | | | | | | | |
| Positive | 14(2.8) | 9(64.3) | 10(3.1) | 7(70.0) | 3(3.0) | 2(66.7) | 1(1.3) | 0.0000 |
| Negative | 81(16.2) | 46(56.8) | 56(17.2) | 37(66.1) | 19(19.2) | 6(31.6) | 6(7.9) | 3(50.0) |
| Unknown | 406(81.0) | 235(57.9) | 260(79.8) | 177(68.1) | 77(77.8) | 28(36.4) | 69(90.8) | 30(43.5) |
| **Neck dissection[a]** | | | | | | | | |
| SOND | 112(22.4) | 54(48.2) | 80(24.5) | 43(53.8) | 18(18.2) | 8(44.4) | 14(18.4) | 3(21.4) |
| Extended SOND | 107(21.4) | 64(60.0) | 74(22.7) | 56(75.7) | 23(23.2) | 6(26.1) | 10(13.2) | 2(20.0) |
| RND | 282(56.3) | 172(61.0) | 172(52.8) | 122(70.9) | 58(58.6) | 22(37.9) | 52(68.4) | 28(53.8) |
| **En-bloc resection** | | | | | | | | |
| Yes | 340(67.9) | 199(58.5) | 252(77.3) | 168(66.7) | 85(85.9) | 29(34.1) | 3(3.9) | 2(66.7) |
| No | 161(32.1) | 91(56.5) | 74(22.7) | 53(71.6) | 14(14.1) | 7(50.0) | 73(96.1) | 31(42.5) |
| **Surgical margin** | | | | | | | | |

| | | | | | | | | |
|---|---|---|---|---|---|---|---|---|
| Positive | 20(4.0) | 4(20.0) | 3(0.9) | 1(33.3) | 13(13.1) | 2(15.4) | 4(5.3) | 1(25.0) |
| Negative | 481(96.0) | 286(59.5) | 323(99.1) | 220(68.1) | 86(86.9) | 34(39.5) | 72(94.7) | 32(44.4) |
| Size of flap (Length of skin island) | | | | | | | | |
| No | 105(21.0) | 64(61.0) | 67(20.6) | 50(74.6) | 11(11.1) | 2(18.2) | 27(35.5) | 12(44.4) |
| 0-10cm | 159(31.7) | 97(61.0) | 101(31.0) | 72(71.3) | 32(32.3) | 12(37.5) | 26(34.2) | 13(50.0) |
| 10-15cm | 131(26.2) | 69(52.7) | 86(26.4) | 53(61.6) | 29(29.3) | 9(31.0) | 16(21.1) | 7(43.8) |
| 15-20cm | 72(14.4) | 37(51.4) | 47(14.4) | 26(55.3) | 22(22.2) | 11(50.0) | 3(3.9) | 0.0000 |
| ⩾20cm | 34(6.8) | 23(67.7) | 25(7.7) | 20(80.0) | 5(5.1) | 2(40.0) | 4(5.3) | 1(25.0) |
| Flap type | | | | | | | | |
| Anterolateral thigh flap | 270(53.9) | 148(54.8) | 191(58.6) | 119(62.3) | 46(46.5) | 14(30.4) | 33(43.4) | 15.0000 |
| Fibular flap | 10(2.0) | 8(80.0) | 10(3.1) | 8(80.0) | 0 | NA | 0.0000 | NA |
| Radial forearm flap | 75(15.0) | 51(68.0) | 53(16.3) | 41(77.4) | 10(10.1) | 4(40.0) | 12(15.8) | 6(50.0) |
| Latissimus dorsi flap | 7(1.4) | 2(28.6) | 1(0.3) | 0.0000 | 5(5.1) | 2(40.0) | 1(1.3) | 0.0000 |
| Pectoralis myocutaneous flap | 34(6.8) | 17(50.0) | 4(1.2) | 3(75.0) | 27(27.3) | 14(51.9) | 3(3.9) | 0.0000 |
| Direct close or regional flap | 105(21.0) | 64(61.0) | 67(20.6) | 50(74.6) | 11(11.1) | 2(18.2) | 27(35.5) | 12.0000 |
| Perioperative complications[&] | | | | | | | | |
| Surgical site infection | 59(11.8) | 24(40.7) | 37(11.4) | 16(43.2) | 12(12.1) | 5(41.7) | 10(13.2) | 3(30.0) |
| Pulmonary infection | 80(16.0) | 46(57.5) | 58(17.8) | 38(65.5) | 9(9.1) | 3(33.3) | 13(17.1) | 5(38.5) |
| Chyle leakage | 19(3.8) | 9(47.4) | 13(4.0) | 6(46.2) | 4(4.0) | 3(75.0) | 2(2.6) | 0.0000 |
| Orocutaneous fistula | 38(7.6) | 14(36.8) | 17(5.2) | 9(52.9) | 11(11.1) | 2(18.2) | 10(13.2) | 3(30.0) |
| Flap necrosis | 17(3.4) | 4(23.5) | 12(3.7) | 4(33.3) | 3(3.0) | 1(33.3) | 2(2.6) | 0.0000 |
| Hematoma | 15(3.0) | 10(66.7) | 11(3.4) | 9(81.8) | 0 | NA | 4(5.3) | 1(25.0) |
| Delirium | 21(4.2) | 10(47.6) | 12(3.7) | 7(58.3) | 7(7.1) | 3(42.9) | 2(2.6) | 0.0000 |
| Wound dehiscence | 44(8.8) | 19(43.2) | 31(9.5) | 14(45.2) | 1(1.0) | 1(100.0) | 12(15.8) | 4(33.3) |
| DVT | 8(1.6) | 6(75.0) | 7(2.1) | 6(85.7) | 0 | NA | 1(1.4) | 0.0000 |
| Postoperative adjuvant therapy | | | | | | | | |
| Radiotherapy | 310(61.9) | 177(57.1) | 202(62.0) | 136(67.3) | 53(53.5) | 16(30.2) | 55(72.4) | 25(45.5) |
| Chemotherapy | 9(1.8) | 4(44.4) | 6(1.8) | 3(50.0) | 3(3.0) | 1(33.3) | 0.0000 | NA |
| Radio-chemotherapy | 100(20.0) | 63(63.0) | 75(23.0) | 54(72.0) | 15(15.2) | 4(26.7) | 10(13.2) | 5(50.0) |
| Radiotherapy and anti-EGFR therapy | 22(4.4) | 13(59.1) | 9(2.8) | 5(55.6) | 9(9.1) | 6(66.7) | 4(5.3) | 2(50.0) |
| Chemotherapy and anti-EGFR therapy | 12(2.4) | 9(75.0) | 10(3.1) | 7(70.0) | 2(2.0) | 2(100.0) | 0.0000 | NA |
| Radio-chemotherapy and anti-EGFR therapy | 24(4.8) | 16(66.7) | 24(7.4) | 16(66.7) | 0 | NA | 0.0000 | NA |
| None | 24(4.8) | 8(33.3) | 0 | NA | 17(17.2) | 7(41.2) | 7(9.2) | 1(14.3) |

NA: Not Applicable

[a]: Neck dissection for the ENE side was counted: SOND: Supra-omohyoid neck dissection (Level I-III); extended SOND: extended supra-omohyoid neck dissection (Level I-IV); RND: Radical neck dissection (Level I-V)

&: Some cases had multiple complications.

HPV: Human papillomavirus; DVT: Deep venous thrombosis.

**Supplementary Table 2. The characteristics of primary or recurrent oral lesions in patients with unilateral ENE nodes**

| Variables | The whole groups | | The PL group | | The RL group | | The INM group | |
|---|---|---|---|---|---|---|---|---|
| | N(%) | The DFS rate | N(%) | The DFS rate | N(%) | The DFS rate | N(%) | The DFS rate |
| **Primary or recurrent subsite** | | | | | | | | |
| Buccal mucosa | 73(14.6) | 38(52.1) | 41(12.6) | 25(12.6) | 11(11.1) | 7(63.6) | 21(27.6) | 6(28.6) |
| Floor of mouth | 137(27.3) | 79(57.7) | 84(25.8) | 61(25.8) | 48(48.5) | 15(31.3) | 5(6.6) | 3(60.0) |
| Retromolar trigone | 20(4.0) | 10(50.0) | 19(5.8) | 10(52.6) | 1(1.0) | 0.0000 | 0.0000 | NA |
| Upper Gingiva | 22(4.4) | 19(86.4) | 16(4.9) | 15(93.8) | 2(2.0) | 1(50.0) | 4(5.3) | 3(75.0) |
| Tongue | 201(40.1) | 113(56.2) | 132(40.5) | 88(66.7) | 31(31.3) | 9(29.0) | 38(50.0) | 16(42.1) |
| Hard palate | 28(65.6) | 18(64.3) | 18(5.5) | 11(61.1) | 3(3.0) | 2(66.7) | 7(9.2) | 5(71.4) |
| Lower Gingiva | 20(4.0) | 13(65.0) | 16(4.9) | 11(68.8) | 3(3.0) | 2(66.7) | 1(1.3) | 0.0000 |
| **Pathologic grade** | | | | | | | | |
| I | 69(13.8) | 35(50.7) | 30(9.2) | 23(76.7) | 20(20.2) | 5.0000 | 19(25.0) | 7(36.8) |
| II | 356(71.1) | 208(58.4) | 245(75.2) | 159(64.9) | 62(62.6) | 25(25.0) | 49(64.5) | 24(49.0) |
| III | 76(15.2) | 47(61.8) | 51(15.6) | 39(76.5) | 17(17.2) | 6(35.3) | 8(10.5) | 2(25.0) |
| **T classification*** | | | | | | | | |
| T1 | 10(2.0) | 7(70.0) | 2(0.6) | 2(100.0) | 4(4.0) | 1(25.0) | 4(5.3) | 4(100.0) |
| T2 | 105(21.0) | 62(59.0) | 67(20.6) | 47(70.2) | 21(21.2) | 7(33.3) | 17(22.4) | 8(47.1) |
| T3 | 284(56.7) | 166(58.5) | 182(55.8) | 127(70.0) | 57(57.6) | 20(35.1) | 45(59.2) | 19(42.2) |
| T4 | 102(20.4) | 55(53.9) | 75(23.0) | 45(60.0) | 17(17.2) | 8(47.1) | 10(13.2) | 2(20.0) |
| **Size of oral lesion** | | | | | | | | |
| 0-2cm | 13(2.6) | 8(61.5) | 5(1.5) | 4(80.0) | 4(4.0) | 1(25.0) | 4(5.3) | 3(75.0) |
| 2-4cm | 165(32.9) | 99(60.0) | 104(31.9) | 74(71.2) | 34(34.3) | 11(32.4) | 27(35.5) | 14(51.9) |
| 4-6cm | 248(49.5) | 145(58.5) | 157(48.2) | 108(68.8) | 53(53.5) | 22(41.5) | 38(50.0) | 15(39.5) |
| >6cm | 75(15.0) | 38(50.7) | 60(18.4) | 35(58.3) | 8(8.1) | 2(25.0) | 7(9.2) | 1(14.3) |
| **DOI>10mm** | | | | | | | | |
| Yes | 239(47.7) | 133(55.6) | 153(46.9) | 100(65.4) | 59(59.6) | 23(39.0) | 27(35.5) | 10(37.0) |
| No | 262(52.3) | 157(60.0) | 173(53.1) | 121(70.0) | 40(40.4) | 13(32.5) | 49(64.5) | 23(46.9) |
| **Midline involvement** | | | | | | | | |
| Yes | 100(20.0) | 53(53.0) | 64(19.6) | 40(62.5) | 20(20.2) | 8(40.0) | 16(21.1) | 5(31.3) |
| No | 401(80.0) | 237(59.1) | 262(80.4) | 181(69.1) | 79(79.8) | 28(35.4) | 60(78.9) | 28(46.7) |
| **PNI** | | | | | | | | |
| Yes | 122(24.4) | 67(54.9) | 82(25.2) | 55(67.1) | 26(26.3) | 9(34.6) | 14(18.4) | 3(21.4) |
| No | 379(75.6) | 223(58.8) | 244(74.9) | 166(68.0) | 73(73.7) | 27(37.0) | 62(81.6) | 30(48.4) |
| **Bone destruction (oral lesion)** | | | | | | | | |
| Yes | 93(18.6) | 43(46.2) | 60(18.4) | 32(53.3) | 23(23.2) | 7(30.4) | 10(13.2) | 4(40.0) |
| No | 408(81.4) | 247(60.5) | 266(81.6) | 189(71.1) | 76(76.8) | 29(38.2) | 66(86.8) | 29(43.9) |

| | | | | | | | | |
|---|---|---|---|---|---|---|---|---|
| **Skull base involvement** | | | | | | | | |
| Yes | 20(4.0) | 8(40.0) | 14(4.3) | 6(42.9) | 5(5.0) | 1(20.0) | 1(1.3) | 1(100.0) |
| No | 481(96.0) | 282(58.6) | 312(95.7) | 215(68.9) | 94(95.0) | 35(37.2) | 75(98.7) | 32(42.7) |

NA: Not Applicable.

*: T classification of the RL or INM group was based on the pathological characteristics of their prior primary lesions according to AJCC system.

**Supplementary Table 3. The characteristics of metastatic lymph nodes and ENE features in patients with unilateral ENE nodes**

| Variables | The whole groups | | The PL group | | The RL group | | The INM group | |
|---|---|---|---|---|---|---|---|---|
| | N(%) | The DFS rate | N(%) | The DFS rate | N(%) | The DFS rate | N(%) | The DFS rate |
| **ENE found via preoperative imaging** | | | | | | | | |
| Yes | 216(43.1) | 98(45.4) | 137(42.0) | 72(52.6) | 44(44.4) | 13(29.5) | 35(46.1) | 13(37.1) |
| No | 285(56.9) | 192(67.4) | 189(58.0) | 149(78.8) | 55(55.6) | 23(41.8) | 41(53.9) | 20(48.8) |
| **ICCR-subclassification** | | | | | | | | |
| ENEma | 108(21.6) | 65(60.2) | 68(20.9) | 49(72.1) | 24(24.2) | 8(33.3) | 16(21.1) | 8(50.0) |
| ENEmi | 393(78.4) | 225(57.3) | 258(79.1) | 172(66.7) | 75(75.8) | 28(37.3) | 60(78.9) | 25(41.7) |
| **Fusion of oral lesion and metastatic lymph node** | | | | | | | | |
| Yes | 2(0.4) | 0.0 | 2(0.6) | 0.0 | 0.0 | NA | 0.0 | NA |
| No | 499(99.6) | 290(58.1) | 324(99.4) | 221(68.2) | 99(100.0) | 36(36.4) | 76(100.0) | 33(43.4) |
| **Maximum size of ENE nodes** | | | | | | | | |
| Mean ± standard deviation | 3.6±1.2 | | 3.6±1.2 | | 3.6±1.3 | | 3.7±1.3 | |
| **Number of lymph nodes** | | | | | | | | |
| Mean ± standard deviation | 21±12.7 | | 21±12.6 | | 21.9±11.8 | | 21.6±14.0 | |
| **Number of metastatic lymph nodes** | | | | | | | | |
| Mean ± standard deviation | 4±4.1 | | 3±4.1 | | 4.9±4.5 | | 3.8±3.3 | |
| **Number of ENE nodes** | | | | | | | | |
| Mean ± standard deviation | 2±2.2 | | 2±2.4 | | 1.9±1.7 | | 2.0±1.8 | |
| **Sides of ENE lymph nodes** | | | | | | | | |
| Ipsilateral | 416(83.0) | 246(59.1) | 279(85.6) | 192(85.6) | 74(74.7) | 27(36.5) | 63(82.9) | 27(42.9) |
| Contralateral | 85(17.0) | 44(51.8) | 47(14.4) | 29(61.7) | 25(25.3) | 9(36.0) | 13(17.1) | 6(46.2) |
| **Level of ENE nodes** | | | | | | | | |
| I-III | 422(84.2) | 244(57.8) | 278(85.3) | 190(68.3) | 82(82.8) | 32(39.0) | 62(81.6) | 22(35.5) |
| IV-V | 48(9.6) | 28(58.3) | 32(9.8) | 20(62.5) | 8(8.1) | 1(12.5) | 8(10.5) | 7(87.5) |
| Both | 31(6.2) | 18(58.1) | 16(5.0) | 11(68.8) | 9(9.1) | 3(33.3) | 6(7.9) | 4(66.7) |
| **Soft tissue involvement** | | | | | | | | |

| | | | | | | | | | |
|---|---|---|---|---|---|---|---|---|---|
| | Yes | 232(46.3) | 121(52.2) | 152(46.6) | 91(59.9) | 42(42.4) | 13(31.0) | 38(50.0) | 17(44.7) |
| | No | 269(53.7) | 169(62.8) | 174(53.4) | 130(74.7) | 57(57.6) | 23(40.4) | 38(50.0) | 16(42.1) |
| **Muscular invasion** | | | | | | | | | |
| | Yes | 246(49.1) | 122(49.6) | 157(48.2) | 91(58.0) | 51(51.5) | 16(31.4) | 38(50.0) | 15(39.5) |
| | No | 255(50.9) | 168(65.9) | 169(51.8) | 130(76.9) | 48(48.5) | 20(41.7) | 38(50.0) | 18(47.4) |
| **Arterial encasement** | | | | | | | | | |
| | Yes | 15(3.0) | 2(13.3) | 10(3.1) | 2(20.0) | 3(3.0) | 0.0 | 2(26.3) | 0.0 |
| | No | 486(97.0) | 288(59.3) | 316(96.9) | 219(69.3) | 96(97.0) | 36(37.5) | 74(97.4) | 33(44.6) |
| **Internal jugular cancer embolism** | | | | | | | | | |
| | Yes | 48(9.6) | 16(33.3) | 29(8.9) | 12(41.4) | 13(13.1) | 2(15.4) | 6(7.9) | 2(33.3) |
| | No | 453(90.4) | 274(60.5) | 297(91.1) | 209(70.4) | 86(86.9) | 34(39.5) | 70(92.1) | 31(44.3) |
| **Lymph node necrosis** | | | | | | | | | |
| | Yes | 132(26.3) | 56(42.4) | 88(27.0) | 44(50.0) | 25(25.3) | 6(24.0) | 19(25.0) | 6(31.6) |
| | No | 369(73.7) | 234(63.4) | 238(73.0) | 177(74.4) | 74(74.7) | 30(40.5) | 57(75.0) | 27(47.4) |
| **Bone involvement** | | | | | | | | | |
| | Yes | 50(10.0) | 25(50.0) | 35(10.7) | 19(54.3) | 9(9.1) | 2(22.2) | 6(7.9) | 4(66.7) |
| | No | 451(90.0) | 265(58.8) | 291(89.3) | 202(69.4) | 90(90.9) | 34(37.8) | 70(92.1) | 29(41.4) |
| **Lymph node ratio (LNR)*** | | | | | | | | | |
| | Mean ± standard deviation | 0.26±0.29 | | 0.26±0.28 | | 0.24±0.24 | | 0.28±0.28 | |
| **Number ratio between ENE and excised nodes&** | | | | | | | | | |
| | Mean ± standard deviation | 0.19±0.27 | | 0.18±0.25 | | 0.14±0.17 | | 0.19±0.25 | |

**NA: Not Applicable**

***: The number of metastatic nodes divided by the total number of excised nodes.**

**&: The number of ENE nodes divided by the total number of excised nodes.**

**Supplementary Table 4. Summary of death causes of patients with unilateral ENE nodes**

| Variables | N(%) | | | |
|---|---|---|---|---|
| | The whole groups | The PL group | The RL group | The INM group |
| **Death causes (Overall)** | | | | |
| locoregional recurrence | 94(44.6) | 53(50.5) | 25(39.7) | 16(27.2) |
| Distant metastasis | 54(25.6) | 29(27.6) | 12(19.0) | 13(30.2) |
| Distant metastasis and locoregional recurrence | 55(26.1) | 20(19.0) | 22(34.9) | 13(30.2) |
| Non-oncologic cause | 8(3.8) | 3(2.9) | 4(6.3) | 1(2.3) |

**Supplementary Table 5. The Cox regression DFS survival analysis of the characteristics of patients with unilateral ENE nodes**

| Variables | The whole groups | | The PL group | | The RL group | | The INM group | |
|---|---|---|---|---|---|---|---|---|
| | Univariate analysis | Multivariate analysis(OR,95%CI) | Univariate analysis | Multivariate analysis(OR,95%CI) | Univariate analysis | Multivariate analysis(OR,95%CI) | Univariate analysis | Multivariate analysis(OR,95%CI) |
| Treatment group | <0.001 | <0.001(1.295~1.785) | NA | | NA | | NA | |
| ENE found via preoperative imaging | <0.001 | <0.001(1.365~2.969) | <0.001 | <0.001(1.917~5.430) | 0.078 | | 0.375 | |
| ICCR-subclassification | 0.437 | | 0.418 | 0.030(0.261~0.935) | 0.612 | | 0.399 | |
| Fusion of oral lesion and metastatic lymph nodes | 0.012 | | 0.003 | 0.018(1.422~40.671) | NA | | NA | |
| Maximum size of ENE nodes | <0.001 | 0.036(0.696~0.988) | <0.001 | | 0.066 | | 0.775 | |
| Number of lymph nodes | 0.255 | | 0.005 | | 0.026 | 0.017(1.004~1.046) | 0.830 | 0.082(0.936~1.004) |
| Number of metastatic lymph nodes | 0.539 | | 0.570 | | 0.301 | | 0.085 | |
| Number of ENE nodes | 0.206 | | 0.037 | | 0.602 | | 0.346 | |
| Level of ENE nodes | 0.974 | | 0.812 | | 0.238 | | 0.040 | |
| The side of the ENE nodes | 0.167 | | 0.343 | | 0.856 | | 0.933 | |
| Soft tissue involvement | 0.012 | | 0.004 | | 0.210 | | 0.634 | |
| Muscular invasion | <0.001 | | <0.001 | | 0.088 | | 0.466 | |
| Arterial encasement | <0.001 | 0.035(1.047~3.584) | <0.001 | 0.010(1.917~5.430) | 0.013 | | 0.475 | |
| Internal jugular cancer embolism | <0.001 | 0.016(1.103~2.616) | 0.001 | | 0.012 | | 0.571 | |
| metastatic node necrosis | <0.001 | 0.010(1.125~2.350) | <0.001 | | 0.017 | 0.011(1.175~3.487) | 0.458 | 0.079(0.893~7.647) |
| Bone involvement | 0.205 | | 0.071 | | 0.142 | | 0.251 | |
| HPV status | 0.066 | | 0.856 | | 0.707 | | 0.920 | |
| LNR | 0.024 | 0.031(0.095~0.891) | <0.001 | | 0.734 | | 0.152 | 0.021(<0.001~0.367) |
| Number ratio between ENE and excised nodes | 0.002 | 0.008(1.486~14.602) | <0.001 | 0.001(1.532~4.820) | 0.844 | | 0.809 | 0.048(1.057~47134.133) |

DFS: Disease-free survival.

NA: Not Applicable

HPV: Human papillomavirus; LNR: Lymph node ratio.

**Supplementary Table 6. The Cox regression TTR analysis of the characteristics of patients with unilateral ENE nodes.**

| Variables | The whole groups | | The PL group | | The RL group | | The INM group | |
|---|---|---|---|---|---|---|---|---|
| | Univariate analysis | Multivariate analysis(OR,95%CI) | Univariate analysis | Multivariate analysis(OR,95%CI) | Univariate analysis | Multivariate analysis(OR,95%CI) | Univariate analysis | Multivariate analysis(OR,95%CI) |
| Treatment Group | <0.001 | <0.001(1.292~1.796) | NA | | NA | | NA | |
| ENE found via preoperative imaging | <0.001 | <0.001(1.397~3.070) | <0.001 | <0.001(1.709~4.667) | 0.049 | | 0.294 | 0.085(0.883~6.846) |
| ICCR-subclassification | 0.303 | | 0.395 | 0.076(0.300~1.062) | 0.448 | | 0.374 | |

| | | | | | | | | |
|---|---|---|---|---|---|---|---|---|
| Fusion of oral lesion and metastatic lymph nodes | 0.047 | | 0.016 | | NA | | NA | |
| Maximum size of ENE nodes | <0.001 | 0.031(0.691~0.982) | <0.001 | | 0.031 | | 0.916 | 0.090(0.408~1.067) |
| Number of lymph nodes | 0.087 | | 0.002 | | 0.163 | | 0.805 | 0.024(0.919~0.994) |
| Number of metastatic lymph nodes | 0.647 | | 0.726 | | 0.320 | 0.056(0.993~1.708) | 0.111 | |
| Number of ENE nodes | 0.210 | | 0.059 | | 0.654 | 0.013(0.366~0.888) | 0.362 | |
| Level of ENE nodes | 0.776 | | 0.757 | | 0.182 | 0.021(1.137~4.760) | 0.061 | |
| Side of ENE nodes | 0.252 | | 0.459 | | 0.943 | | 0.978 | |
| Soft tissue involvement | 0.021 | | 0.005 | | 0.331 | | 0.709 | |
| Muscular invasion | <0.001 | | 0.001 | | 0.077 | | 0.533 | |
| Arterial encasement | <0.001 | | <0.001 | <0.001(2.002~8.561) | 0.023 | | 0.827 | 0.094(0.014~1.397) |
| Internal jugular cancer embolism | <0.001 | 0.001(1.332~3.056) | <0.001 | | 0.004 | 0.008(1.307~6.200) | 0.447 | |
| metastatic node necrosis | <0.001 | | <0.001 | | 0.010 | | 0.545 | 0.027(1.154~11.080) |
| Bone involvement | 0.223 | | 0.092 | | 0.176 | | 0.296 | |
| HPV status | 0.697 | | 0.732 | | 0.535 | | 0.920 | |
| LNR | 0.018 | 0.005(0.092~0.872) | <0.001 | | 0.911 | 0.047(<0.001~0.912) | 0.168 | 0.025(<0.001~0.397) |
| Number ratio between ENE and excised nodes | 0.002 | 0.007(1.530~15.379) | <0.001 | 0.001(1.560~4.944) | 0.953 | | 0.715 | |

**TTR: Time-to-relapse.**
**NA: Not Applicable.**
**HPV: Human papillomavirus; LNR: Lymph node ratio.**

**Supplementary Table 7. The Cox regression TTR analysis of demographics and treatment in patients with bilateral ENE nodes**

| Variables | The whole groups | | The PL group | | The RL group | | The INM group | |
|---|---|---|---|---|---|---|---|---|
| | Univariate Analysis | Multivariate analysis(OR,95%CI) | Univariate Analysis | Multivariate analysis(OR,95%CI) | Univariate Analysis | Multivariate analysis(OR,95%CI) | Univariate Analysis | Multivariate analysis(OR,95%CI) |
| Treatment group | 0.038 | | | | | | | |
| Age | 0.301 | | 0.746 | | 0.364 | | 0.572 | |
| Sex | 0.622 | | 0.843 | | 0.640 | | 0.716 | |
| Histories of smoking and alcohol | 0.297 | | 0.631 | | 0.658 | | 0.445 | |
| Comorbidities | 0.310 | | 0.252 | | 0.026 | | 0.418 | |
| History of prior treatment | 0.079 | | 0.150 | | NA | | 0.723 | |
| Ipsilateral neck dissection | 0.702 | | 0.379 | | 0.861 | | 0.882 | |
| Contralateral neck dissection | 0.509 | | 0.459 | | 0.586 | | 0.592 | |
| En-bloc resection | 0.067 | | 0.097 | 0.032(0.287~0.946) | 0.428 | | NA | |

| | | | | | | | |
|---|---|---|---|---|---|---|---|
| Surgical margin | 0.008 | 0.006(1.322~5.114) | 0.258 | | 0.019 | 0.019(1.270~13.884) | 0.390 |
| Flap size | 0.150 | | 0.373 | | 0.932 | | 0.077 |
| Flap type | 0.892 | | 0.625 | | 0.507 | | 0.087 |
| Postoperative adjuvant therapy | 0.018 | 0.020(0.727~0.974) | 0.880 | | 0.450 | | 0.338 |
| HPV status | 0.725 | | 0.719 | | 0.549 | | 0.713 |
| Perioperative complications | 0.018 | 0.047(1.006~2.485) | 0.007 | 0.003(1.375~4.666) | 0.542 | | 0.172 |

TTR: Time-to-relapse.

NA: Not applicable.

*: Including a case whose primary lesions were found both in the tongue and thyroid, but mostly the tongue.

HPV: Human papillomavirus.

**Supplementary Table 8. The Cox regression TTR analysis of metastatic lymph nodes in patients with bilateral ENE nodes.**

| Variables | The whole groups | | The PL group | | The RL group | | The INM group | |
|---|---|---|---|---|---|---|---|---|
| | Univariate analysis | Multivariate analysis(OR,95%CI) | Univariate analysis | Multivariate analysis(OR,95%CI) | Univariate analysis | Multivariate analysis(OR,95%CI) | Univariate analysis | Multivariate analysis(OR,95%CI) |
| ENE found via preoperative imaging | 0.079 | | 0.698 | | 0.261 | | 0.210 | |
| ICCR-subclassification | 0.005 | 0.060(0.966~5.585) | 0.017 | | 0.351 | | 0.308 | |
| Fusion of oral lesion and metastatic lymph nodes | 0.194 | | 0.108 | | 0.282 | | 0.159 | |
| Maximum size of ENE nodes | <0.001 | 0.039(1.013~1.661) | 0.003 | | 0.540 | | 0.009 | 0.004(1.243~3.058) |
| Number of ipsilateral lymph nodes | 0.621 | | 0.604 | 0.072(0.996~1.101) | 0.515 | | 0.110 | |
| Number of contralateral lymph nodes | 0.397 | | 0.819 | | 0.257 | | 0.690 | |
| Number of ipsilateral metastatic lymph nodes | 0.774 | | 0.794 | | 0.233 | 0.013(0.666~0.952) | 0.281 | |
| Number of contralateral metastatic lymph nodes | 0.849 | | 0.578 | | 0.069 | | 0.099 | 0.099(0.975~1.334) |
| Number of ipsilateral ENE nodes | 0.631 | | 0.358 | | 0.685 | | 0.885 | |
| Number of contralateral ENE nodes | 0.500 | | 0.262 | | 0.556 | | 0.091 | |
| Level of ipsilateral ENE nodes | 0.956 | | 0.498 | | 0.046 | | 0.432 | |
| Level of contralateral ENE nodes | 0.866 | | 0.294 | | 0.812 | | 0.562 | |
| Bilateral ENE nodes in I-III level | 0.947 | | 0.725 | | 0.009 | 0.001(2.228~20.259) | 0.432 | |
| Fusion of ipsilateral metastatic lymph nodes | 0.329 | | 0.195 | | 0.697 | | 0.797 | |
| Fusion of contralateral metastatic lymph nodes | 0.261 | | 0.693 | | 0.887 | | 0.317 | |
| Soft tissue involvement | 0.007 | | 0.020 | 0.055(0.006~0.060) | 0.092 | | 0.201 | |
| Muscular invasion | 0.004 | | 0.004 | 0.016(0.336~17.346) | 0.115 | | 0.185 | |
| Arterial encasement | 0.001 | 0.092(0.957~1.806) | 0.044 | 0.010(1.258~5.344) | 0.223 | | 0.042 | |
| Internal jugular cancer embolism | 0.045 | | 0.718 | 0.054(0.013~1.037) | 0.002 | <0.001(8.582~1305.690) | 0.050 | |
| metastatic node necrosis | 0.630 | | 0.845 | | 0.007 | | 0.977 | |
| Bone involvement | 0.056 | | 0.084 | | 0.668 | | 0.494 | |
| HPV status | 0.725 | | 0.719 | | 0.549 | | 0.713 | |
| LNR | 0.53 | | 0.955 | | 0.085 | | 0.02 | |

| | | | | |
|---|---|---|---|---|
| **Number ratio between ENE and excised nodes** | **0.114** | **0.834** | **0.837** | **0.118** |

**NA: Not Applicable.**
